# Differential changes in end organ immune cells and inflammation in salt-sensitive hypertension: effects of lowering blood pressure

**DOI:** 10.1042/CS20240698

**Published:** 2024-07-16

**Authors:** Shobana Navaneethabalakrishnan, Bethany L. Goodlett, Hannah L. Smith, Alyssa Cardenas, Asia Burns, Brett M. Mitchell

**Affiliations:** Department of Medical Physiology, Texas A&M University College of Medicine, Bryan, TX, U.S.A.

**Keywords:** hypertension, lymphatics, macrophages, ovaries, renal physiology, testes

## Abstract

We reported that salt-sensitive hypertension (SSHTN) is associated with increased pro-inflammatory immune cells, inflammation, and inflammation-associated lymphangiogenesis in the kidneys and gonads of male and female mice. However, it is unknown whether these adverse end organ effects result from increased blood pressure (BP), elevated levels of salt, or both. We hypothesized that pharmaceutically lowering BP would not fully alleviate the renal and gonadal immune cell accumulation, inflammation, and lymphangiogenesis associated with SSHTN. SSHTN was induced in male and female C57BL6/J mice by administering nitro-L-arginine methyl ester hydrochloride (L-NAME; 0.5 mg/ml) in their drinking water for 2 weeks, followed by a 2-week washout period. Subsequently, the mice received a 3-week 4% high salt diet (SSHTN). The treatment group underwent the same SSHTN induction protocol but received hydralazine (HYD; 250 mg/L) in their drinking water during the diet phase (SSHTN+HYD). Control mice received tap water and a standard diet for 7 weeks. In addition to decreasing systolic BP, HYD treatment generally decreased pro-inflammatory immune cells and inflammation in the kidneys and gonads of SSHTN mice. Furthermore, the decrease in BP partially alleviated elevated renal and gonadal lymphatics and improved renal and gonadal function in mice with SSHTN. These data demonstrate that high systemic pressure and salt differentially act on end organ immune cells, contributing to the broader understanding of how BP and salt intake collectively shape immune responses and highlight implications for targeted therapeutic interventions.

## Introduction

Hypertension (HTN) plays a significant role in causing end organ damage and failure and stands as a leading factor in premature mortality worldwide [1–3]. Approximately half the adult population is diagnosed with HTN and ∼50% of them exhibit salt-sensitive HTN (SSHTN) [4]. Studies have shown that SSHTN increases the risk of developing health issues, including myocardial infarction, stroke, renal and heart failure, and reproductive health impairments [5–7]. Despite advancements in pharmacological interventions, the effectiveness of anti-hypertensive drugs on SSHTN remains notably limited. This emphasizes the need for further investigations to elucidate pathophysiological mechanisms of SSHTN and develop potential therapeutics specifically tailored for salt-sensitive populations.

Excessive dietary salt intake has been identified as a key factor that raises blood pressure (BP), contributing to the onset of HTN and its associated complications [8,9]. However, the severity of the BP response to salt varies significantly among individuals, categorizing them into salt-sensitive and salt-resistant populations [9]. Extensive research has attempted to unravel the mechanisms underlying this variability in BP response [10,11]. While various factors, such as genetics, environment, race/ethnicity, age, gender, body mass index, and diet, are known contributors to salt sensitivity, the pathophysiological mechanism of SSHTN remains complex and not entirely understood [12]. It is now acknowledged that the renin–angiotensin–aldosterone system, kallikrein–kinin system, sympathetic nervous system, endothelial dysfunction, oxidative stress, and immune system are implicated in the development and progression of SSHTN [13,14]. Mounting evidence from studies involving both humans and animals highlights the crucial role of innate and adaptive immunity in the pathogenesis of SSHTN and associated end organ damage [15–17]. Furthermore, research indicates that a high salt diet directly impacts immune cells, eliciting a pro-inflammatory immune response [18–24]. There is extensive documentation indicating that high salt promotes the activation and functionality of pro-inflammatory M1 macrophages, while concurrently inhibiting the activation and functionality of anti-inflammatory M2 macrophages [18,25–27]. Moreover, increased sodium influx into dendritic cells (DCs) through amiloride-sensitive channels leads to a subsequent increase in isolevuglandin adduct formation and interleukin (IL)-1b synthesis. When activated by elevated salt levels, these DCs further contribute to immune modulation by promoting the production of IL-17a and interferon (IFN) g by T cells [23]. Notably, elevated sodium levels have also been reported to detrimentally affect the function of regulatory T cells (Tregs), while in parallel augmenting the secretion of IFNg and IL-17a from pro-inflammatory T cells, mediated by serum and glucocorticoid-inducible kinase 1 [28,29]. Together, these findings emphasize that elevated salt directly influences immune cells, triggering a cascade of events that contribute to the production of pro-inflammatory cytokines, ultimately modulating the immune response. Nevertheless, the intricate interplay between high salt, BP, and inflammation in HTN remains to be fully elucidated.

Our laboratory and others have reported previously on the infiltration of pro-inflammatory immune cells, inflammation, and inflammation-associated lymphangiogenesis in the kidneys of mice with SSHTN [22,23,26,30,31]. Recently, our group demonstrated that SSHTN also induces an increase in pro-inflammatory macrophages, inflammation, and inflammation-associated lymphangiogenesis in the gonads of male and female mice and this is associated with reproductive dysfunction [7]. However, the specific contributions of high BP, high salt, or both to these adverse effects in the kidneys and gonads remain largely unknown. To address this question, we hypothesized that lowering BP would not completely reverse the renal and gonadal inflammation and end organ damage associated with SSHTN. Using a mouse model of SSHTN, our investigation aimed to elucidate the effects of BP on immune cell populations, inflammation, and lymphatics in the kidneys and gonads of male and female mice with SSHTN.

## Methods

### Animal models

Wild type C57BL/6J mice were purchased from Jackson Laboratories (Bar Harbor, ME) at age 8–10 weeks and acclimatized to our facility for 2 weeks. To induce SSHTN, male and female mice were given nitro-l-arginine methyl ester hydrochloride (L-NAME; 0.5 mg/ml; Sigma, St. Louis, MO) in their drinking water for 2 weeks, followed by a 2-week washout period, after which they received a 4% salt diet (Teklad Envigo, Huntingdon, United Kingdom) for 3 weeks [31,32]. In addition to receiving HTN stimuli, a group of mice also received hydralazine (HYD; 250 mg/L; Sigma) in their drinking water during the 3-week high salt diet phase (SSHTN+HYD). Mice in the control group received a standard chow diet and tap water. All water and diets were provided *ad libitum*. Each group consisted of 14 male and female mice, with 6 allocated to flow cytometry and 8 to other biochemical assays (PCR, sperm parameters, and immunofluorescence). At the conclusion of the 7-week model, mice were killed by exsanguination under 5% inhaled isoflurane anesthesia, with death confirmed by cervical dislocation.

### BP measurements

Systolic BP (SBP) was recorded weekly via tail cuff using the IITC Life Science non-invasive BP acquisition system (IITC Inc., Woodland Hills, CA). All mice were trained in the procedure for 3 days prior to the first reading. Mice were acclimatized to a designated quiet area for half an hour before being transferred to pre-warmed restrainers and warming chambers (34°C). Once in restrainers and tail cuffs, mice acclimated for an additional 5–10 min prior to SBP readings. SBP measurements were determined based on BP tracings by two independent, blinded investigators.

### Flow cytometry

Kidneys were decapsulated, minced thoroughly, and added to digestion buffer containing collagenase D (2.5 mg/ml; Sigma) and dispase II (1 mg/ml; Sigma). Testes and ovaries were minced and placed in digestion buffer containing collagenase II (1 mg/ml; Worthington Biochemicals, Lakewood, NJ), DNAase I (0.15 mg/ml; Sigma), and dispase II (1 mg/ml; Sigma). All tissue samples were incubated at 37°C for 30 min with constant disruption using a gentleMACS Octo Dissociator with heaters (Miltenyi Biotec, Bergisch Gladbach, Germany). Following digestion, single cell suspensions of kidneys, testes, and ovaries were passed through sterile 100 and 40 μm strainers and rinsed with Dulbecco’s phosphate-buffered saline (DPBS, Thermo Fisher Scientific, Waltham, MA). Red blood cells were lysed using ACK lysing buffer (Thermo Fisher Scientific) and rinsed twice with DPBS prior to plating. Cells from each tissue were divided into thirds by volume and assigned to one of three antibody panels (See Online Table I for descriptive panels). They were then incubated with Ghost Dye Violet 510, Ghost Dye Red 710 (Tonbo Biosciences, San Diego, CA), or Zombie Red Fixable Viability Kit (BioLegend, Inc., San Diego, CA), depending on antibody panel assignment, for 30 min on ice. After a DPBS wash, cells were resuspended in 0.1% fetal bovine serum (FBS) solution and incubated with an anti-mouse CD16/CD32 antibody (BD Biosciences, San Jose, CA) for 10 min on ice to block non-specific Fc binding. Next, cells were stained using fluorescent-conjugated antibodies against either CD45, CD11b, CD11c, F4/80, and CD206; CD3e and CD161; or CD45, CD4, and CD25 at a 1:50 dilution for testes and 1:100 for kidneys and ovaries. Cells in the panel containing CD4 underwent additional intracellular staining: they were permeabilized and fixed using a FoxP3 Transcription Factor Staining Buffer Set (Invitrogen, Waltham, MA) and stained with antibodies against IFNg, IL-4, FoxP3, TNFa, and IL-17 on ice for 30 min using the same antibody dilutions noted above. All cells were washed, resuspended in 0.1% FBS solution, and passed through sterile 35 μm strainers prior to flow cytometric analysis. Data was acquired using a BD LSR Fortessa X-20 flow cytometer with FACS DIVA software v9.0 (BD Biosciences). Populations of up to 500,000 cells were analyzed using FlowJo v10.8 (FlowJo, LLC, Ashland, OR). Results are expressed as percentages of respective parent populations. Immune cell subsets were characterized by the markers as described in Online Table II. Gating strategies are displayed in Supplementary Figures S1–9 and were justified by referencing unstained specimens and compensation controls.

### Real-time quantitative PCR

Decapsulated kidneys, testes, and ovaries were frozen in liquid nitrogen prior to storage at −80°C. Total RNA was isolated using a Quick-RNA Miniprep Kit (Zymo Research, Irvine, CA), following the manufacturer’s instructions. Approximately 1 µg of RNA was reverse transcribed into cDNA using an RT^2^ First Strand Kit (Qiagen, Germantown, MD), following the manufacturer’s protocol. Reactions of 10 µl volume were prepared by mixing PowerUp SYBR Green Master Mix (Applied Biosystems, Waltham, MA), nuclease-free water (Invitrogen), and gene primers (10 μM; Sigma), along with cDNA from the relevant tissue type. Expression of mRNA was determined by real-time quantitative PCR (qRT-PCR) using a QuantStudio6 Flex Real-Time PCR system (Applied Biosystems, Foster City, CA). Fold changes of gene expression were calculated using the 2^−ΔΔCT^ method and ubiquitin as an endogenous control. All primers were designed using the NCBI Gene Database and are listed in Online Table III.

### Immunofluorescence

Kidneys, testes, and ovaries were fixed in 4% PFA (Sigma) at 4°C for 24 h. Following fixation, tissues were rinsed with DPBS, embedded in paraffin, and cut into 5 μm sections. Kidneys were cut sagittally and gonadal tissues were cross-sectioned. Tissue sections were deparaffinized, rehydrated, and permeabilized with 0.1% Triton solution (Bio-Rad Laboratories, Inc., Hercules, CA). Sections were then incubated with 10% AquaBlock (EastCoastBio, North Berwick, ME) for 1 h at room temperature to prevent non-specific binding. Subsequently, sections were immunolabeled with LYVE1 (goat polyclonal; R&D Systems, Minneapolis, MN) to identify lymphatic endothelial cells and PECAM-1 (rabbit polyclonal; Abcam, Cambridge, U.K.) to identify endothelial cells. After incubating overnight at 4°C in primary antibodies, sections were washed and incubated with Alexa Fluor 488 and 594 antibodies (Invitrogen) at room temperature for 1 h. After visualization was confirmed, tissues were mounted to slides using Prolong Gold antifade reagent with DAPI (Invitrogen). Images were acquired using an Olympus BX51 fluorescence microscope equipped with a DP72 camera (Olympus, Shinjuku, Tokyo, Japan). Images were captured at 4× for ovaries, 10× for testes, and 20× for kidneys using cellSens Standard software v1.9 (Olympus).

For quantification of LYVE1+ lymphatic vessels, blinded investigators took images of pre-determined areas of each tissue type. For each kidney section, 5–8 cortical images were taken at 20×. Tissue defects, minor calyx, and large numbers of glomeruli were avoided. For testes sections, 4–5 images were captured at 10× in the outer tunica albuginea area. For ovaries, images were taken of the whole tissue at 4×. The total number of LYVE1+ pixels per image was calculated using ImageJ software v1.54 (NIH, Rockville, MD) by establishing a threshold for positive endothelium.

### Serum and urine collection and measures

At the end of the 3 week high salt diet, mice were allowed to acclimate overnight in single capacity metabolic collection cages (Hatteras, Cary, NC). The following morning, urine collection tubes were replaced, and urine was collected continuously over a 24-h period just before euthanization. Blood was obtained via the left ventricle and serum was isolated and stored along with the urine samples at −80°C until analysis. Sodium concentrations in serum and urine were analyzed by capillary electrophoresis using a DxC 700 AU Chemistry Analyzer (Beckman Coulter, Brea, CA). Creatinine concentrations in serum and urine were analyzed by direct potentiometry using a P/ACE MDQ Plus Capillary Electrophoresis System (Sciex, Redwood City, CA). These measurements were utilized to calculate urinary excretion and fractional excretion of sodium (FENa).

### Sperm concentration and morphology

Caudal epididymides from each mouse were placed on pre-warmed (37°C) Biggers-Whitten-Whittingham media and punctured using sterile needles to facilitate the release of spermatozoa. After incubating for 10 min, sperm were loaded into a Neubauer chamber (Sigma) and examined under an Olympus BX51 microscope (Olympus) at 400×. Sperm count was determined and expressed in million sperm/ml.

To assess sperm morphology, thin layers of respective sperm suspensions were diluted to 1:20, smeared on to clean slides, fixed with 95% ethanol, and air dried. The slides were stained with hematoxylin-eosin and 200 spermatozoa from each mouse were analyzed at 400× magnification using an Olympus BX51 microscope (Olympus). Sperm anomalies were recorded and expressed in percentages.

### Sperm acrosome integrity and mitochondrial activity

Sperm acrosome integrity was assessed as described previously [33]. Diluted (1:20) sperm suspensions were smeared onto clean microscope slides, fixed for 15 min in methanol, and air-dried. The slides were then treated with fluorescent-labeled peanut agglutinin (60 µg/mL; Sigma) in the dark for 30 min to specifically label the outer acrosomal membrane. Excess stain was removed by rinsing with Milli-Q water. A total of 200 spermatozoa were evaluated for each mouse under an Olympus BX51 fluorescence microscope (Olympus) at 400× magnification. Acrosomes were classified as intact or damaged based on the observed staining pattern.

To evaluate sperm mitochondrial activity, 1 mL portions of respective diluted (1:20) sperm suspensions underwent staining with 5 µl of Rhodamine 123 (1 mg/ml; Sigma), which is taken up by functional mitochondria. The stained samples were incubated in the dark at 25°C for 10 min and subsequently centrifuged at 300 × ***g*** for 10 min. The resulting pellets were re-suspended in 1 ml phosphate-buffered saline (PBS), mounted onto glass slides, and observed at 400× magnification using an Olympus BX51 fluorescence microscope equipped with a DP72 camera (Olympus). A total of 200 spermatozoa were examined per animal and results were expressed as percentages of mitochondrial activity.

### Statistics

Statistical analyses were conducted using GraphPad Prism version 8.4.3 (GraphPad Software, Inc., Boston, MA). Results are depicted in dot plots or bar graphs showing mean ± SEM. Differences between groups were evaluated through one-way analysis of variance (ANOVA), followed by a Tukey’s post-hoc analysis to examine differences between groups, and the statistical significance defined at *P*<0.05.

### Study approval

All animal procedures performed in mice were approved by the Texas A&M University IACUC in accordance with the *NIH Guide for the Care and Use of Laboratory Animals*.

## Results

### HYD treatment successfully decreased SBP and was associated with decreased renal pro-inflammatory immune cells and inflammation in male SSHTN mice

**Figure 1:**
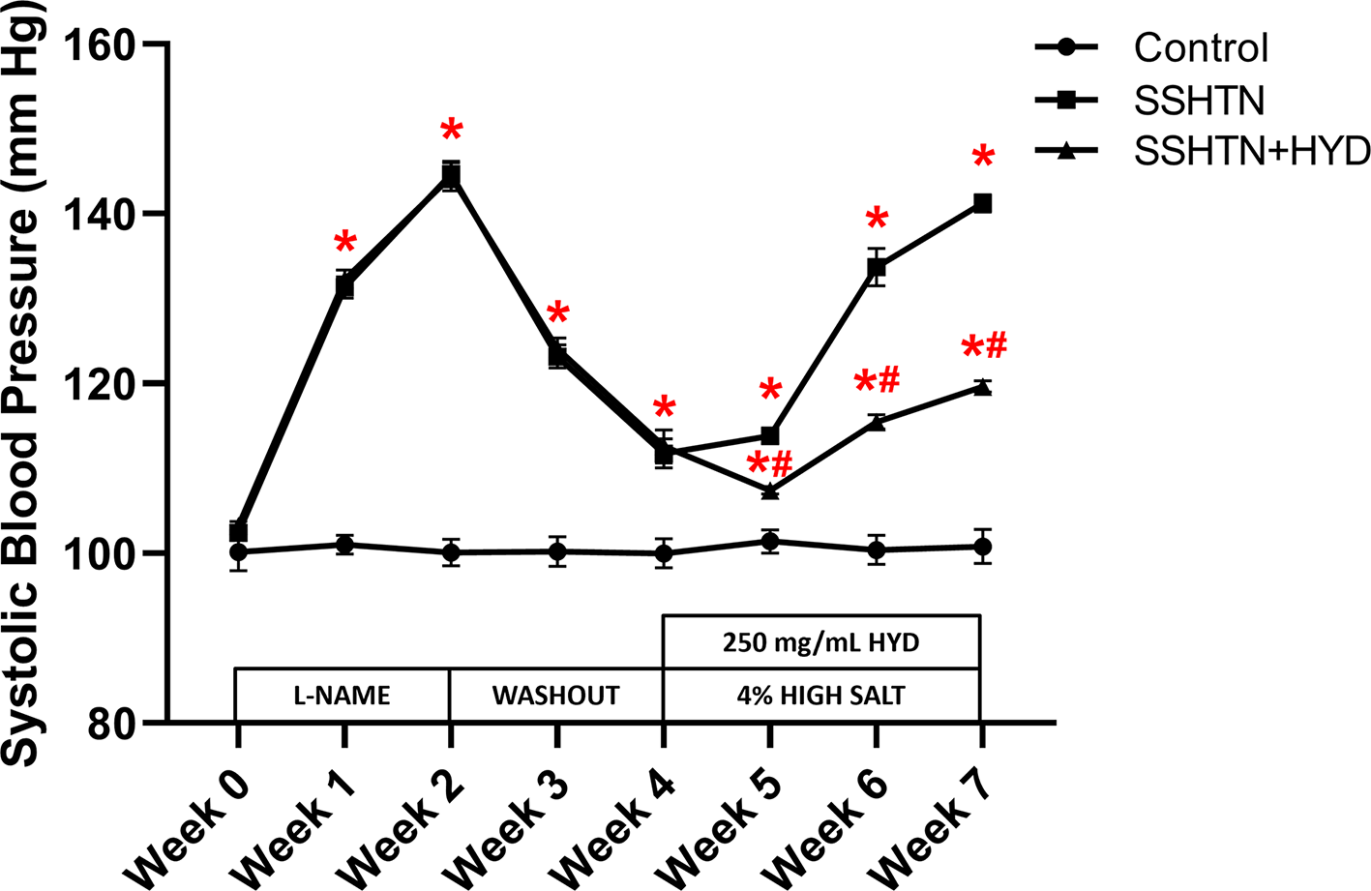
Hydralazine treatment attenuated SBP in male mice with SSHTN SBP measurements in untreated control, SSHTN, and hydralazine (HYD)-treated SSHTN male mice. Blood pressures were taken weekly via tail cuff. Results are presented as mean ± SEM and statistical analyses were performed with a one-way ANOVA (*n* = 5 per group). *P<0.05 vs control mice and #*P*<0.05 vs SSHTN mice.

Male SSHTN mice were administered HYD in the drinking water along with a high salt diet during the last 3 weeks of treatment. SBP was significantly increased in both SSHTN and SSHTN+HYD mice compared with control mice throughout the duration of the model (Figure 1). However, SSHTN+HYD mice had a significantly decreased SBP each week of the diet phase when compared with SSHTN mice (Figure 1).

**Figure 2:**
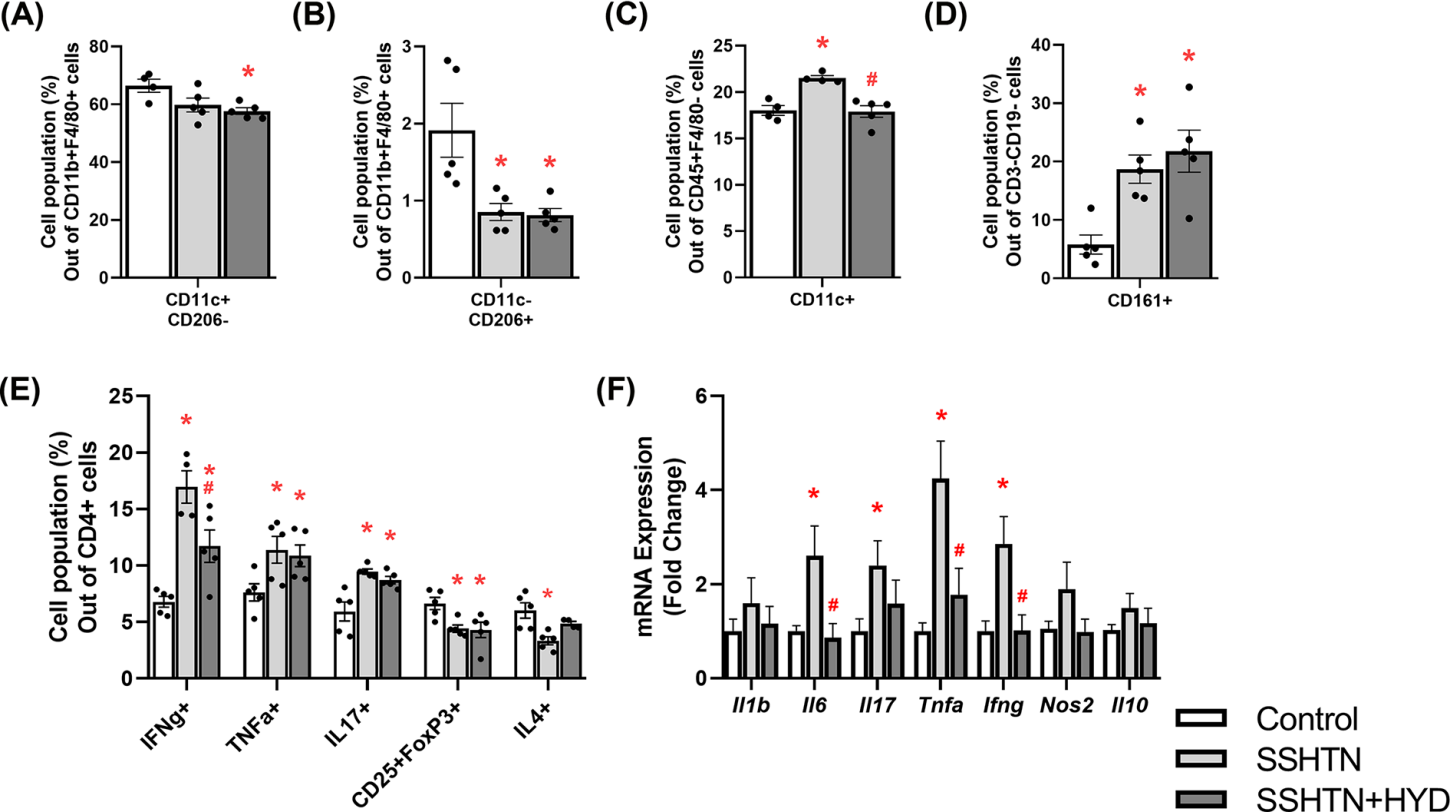
Hydralazine treatment differentially altered immune cells and inflammation in the kidneys of male mice with SSHTN Flow cytometric data examining renal populations of (**A**) M1 macrophages, (**B**) M2 macrophages, (**C**) DCs, (**D**) NK cells, and (**E**) T cells in control, SSHTN, and SSHTN+HYD male mice. Each cell population is shown as a percentage of its respective parent population. (**F**) Renal cytokine expression changes in control, SSHTN, and SSHTN+HYD male mice. Results are presented as mean ± SEM and statistical analyses were performed with one-way ANOVA (*n* = 4−6 per group). *P<0.05 vs control mice and #*P*<0.05 vs SSHTN mice.

To study the effects of decreased BP on renal immune cells, we performed flow cytometry and found SSHTN+HYD mice had a decrease in renal pro-inflammatory M1 macrophages when compared with control mice (Figure 2A). There was a significant decrease in renal anti-inflammatory M2 macrophages in both SSHTN and SSHTN+HYD mice when compared with control mice (Figure 2B). Renal DCs were significantly increased in SSHTN mice and HYD treatment prevented this increase in SSHTN+HYD mice (Figure 2C). There was a significant increase in natural killer (NK) cells in the kidneys of both SSHTN and SSHTN+HYD mice when compared with control mice (Figure 2D). We also found significant increases in renal CD4+IFNg+ and CD4+TNFa+ T helper 1 (Th1) cells, along with increased CD4+IL17+ T helper 17 (Th17) cells in both SSHTN and SSHTN+HYD mice when compared with controls (Figure 2E). HYD treatment decreased CD4+IFNg+ Th1 cells in SSHTN+HYD mice compared with SSHTN mice (Figure 2E). There was a decrease in CD4+CD25+FoxP3+ Tregs in the kidneys of both SSHTN and SSHTN+HYD mice (Figure 2E). Renal CD4+IL4+ T helper 2 (Th2) cells were also decreased in SSHTN mice compared with control mice; however, these Th2 cells were not changed in SSHTN+HYD mice compared with controls (Figure 2E). These findings highlight the differential effects on immune cells (pressure vs salt) in male kidneys following BP reduction, revealing a complex interplay between SSHTN and the immune response.

To further investigate the effects of lowered BP on renal inflammation, we analyzed the gene expression of various cytokines by qRT-PCR. There was a significant increase in the expression of *Il6, Il17, Tnfa*, and *Ifng* in the kidneys of SSHTN mice compared with kidneys from control mice (Figure 2F). On the contrary, there was a significant decrease in *Il6, Tnfa*, and *Ifng* expression in kidneys from SSHTN+HYD mice compared with kidneys from SSHTN mice (Figure 2F). There were no significant changes in *Il1b, Nos2*, and *Il10* expression in the kidneys of either SSHTN or SSHTN+HYD mice in reference to control kidneys (Figure 2F).

### HYD treatment attenuated renal lymphangiogenesis in male SSHTN mice

**Figure 3:**
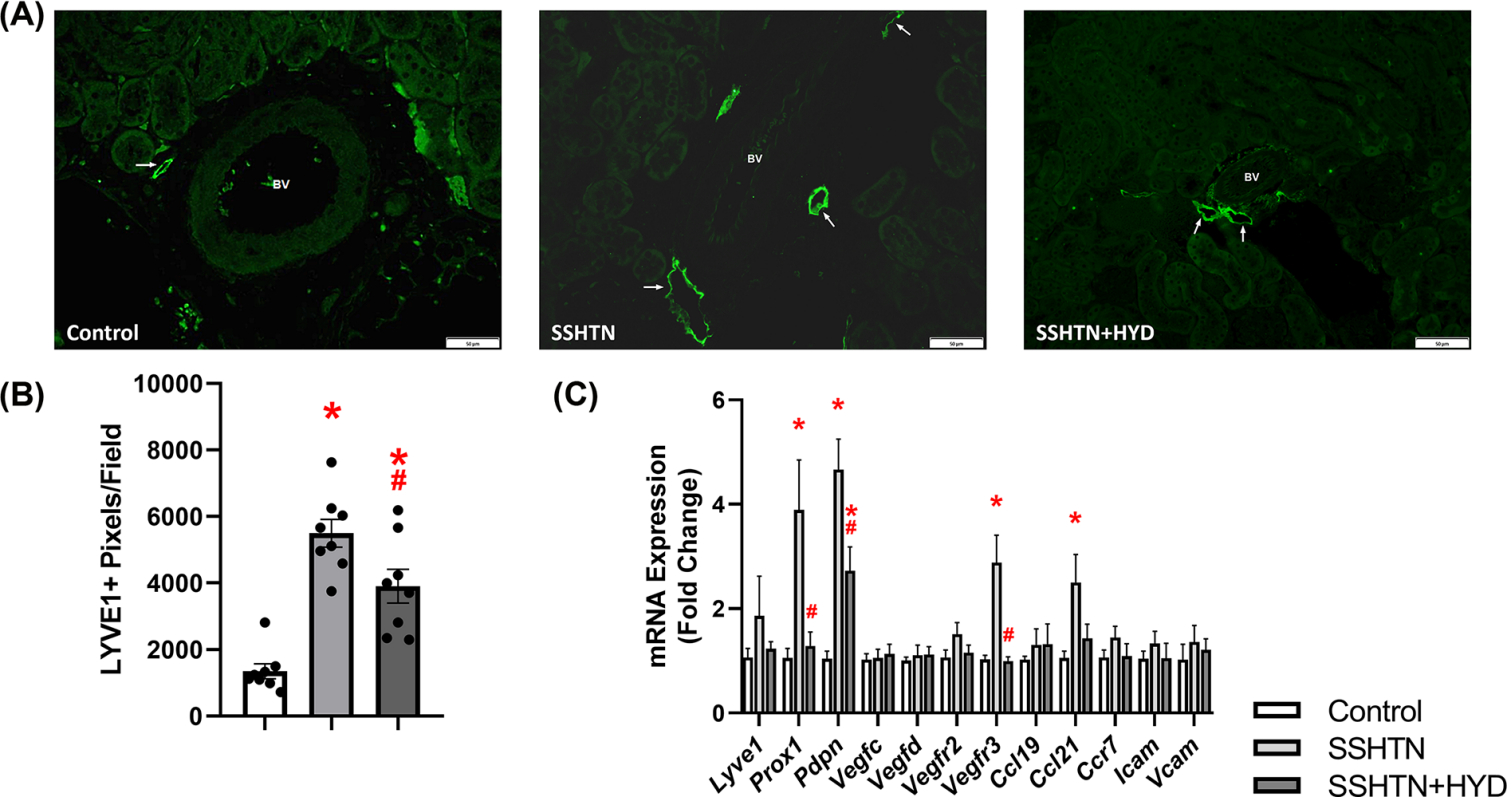
Hydralazine treatment decreased lymphatics in the kidneys of male mice with SSHTN (**A**) LYVE1 immunofluorescence in the kidneys of control, SSHTN, and SSHTN+HYD male mice. LYVE1 is labelled green and lymphatic vessels are indicated with arrows surrounding the blood vessels (BV). Images were taken in the cortex at 20×. Scale bars = 50 µm. (**B**) Renal lymphatic density in control, SSHTN, and SSHTN+HYD male mice as determined by quantification of LYVE1+ pixels per field (*n* = 8 per group). (**C**) Renal expression changes in lymphangiogenesis-related genes in control, SSHTN, and SSHTN+HYD male mice (*n* = 4−5 per group). Results are presented as mean ± SEM and all statistical analyses were performed with one-way ANOVA. *P<0.05 vs control mice and #*P*<0.05 vs SSHTN mice.

Our lab and others have confirmed previously that SSHTN promotes inflammation and inflammation-associated lymphangiogenesis in the kidney [16,17,30,31]. To investigate whether HYD treatment could attenuate the increased lymphatic density associated with SSHTN, we immunolabelled kidney sections with LYVE1, a lymphatic vessel marker. Quantification of LYVE1+ pixels per field revealed a significant increase in renal lymphatic density in SSHTN and SSHTN+HYD mice when compared with control mice (Figure 3A,B). Compared with the SSHTN group, SSHTN+HDZ mice had significantly decreased renal lymphatic density, indicating that HYD treatment partially attenuated renal lymphangiogenesis (Figure 3A,B). To further explore this finding, we analyzed gene expression of lymphatic vessel markers, chemokines, and relevant receptors. In the kidneys of male SSHTN mice, there was an increase in the expression of lymphatic endothelial cell transcription factor *Prox1*, lymphatic endothelial cell marker *Pdpn*, lymphangiogenic growth factor receptor *Vegfr3*, and lymphatic-specific chemokine *Ccl21* when compared with the kidneys of control mice (Figure 3C). HYD treatment significantly decreased renal expression of *Prox1* and *Vegfr3* in SSHTN+HYD mice, bringing these values closer to those of untreated controls (Figure 3C). Renal *Ccl21* expression did not increase in SSHTN+HYD mice, as it had in SSHTN mice (Figure 3C). Additionally, *Pdpn* expression was significantly decreased in the kidneys of SSHTN+HYD mice compared with those of SSHTN mice; however, SSHTN+HYD mice still had significantly increased renal *Pdpn* expression compared with control mice (Figure 3C). Together, these results demonstrate that renal lymphatic density in SSHTN mice was decreased, but not fully mitigated by BP reduction, underscoring the crucial role of high salt in triggering inflammation-associated lymphangiogenesis.

### HYD treatment improved renal function in male SSHTN mice

**Figure 4:**
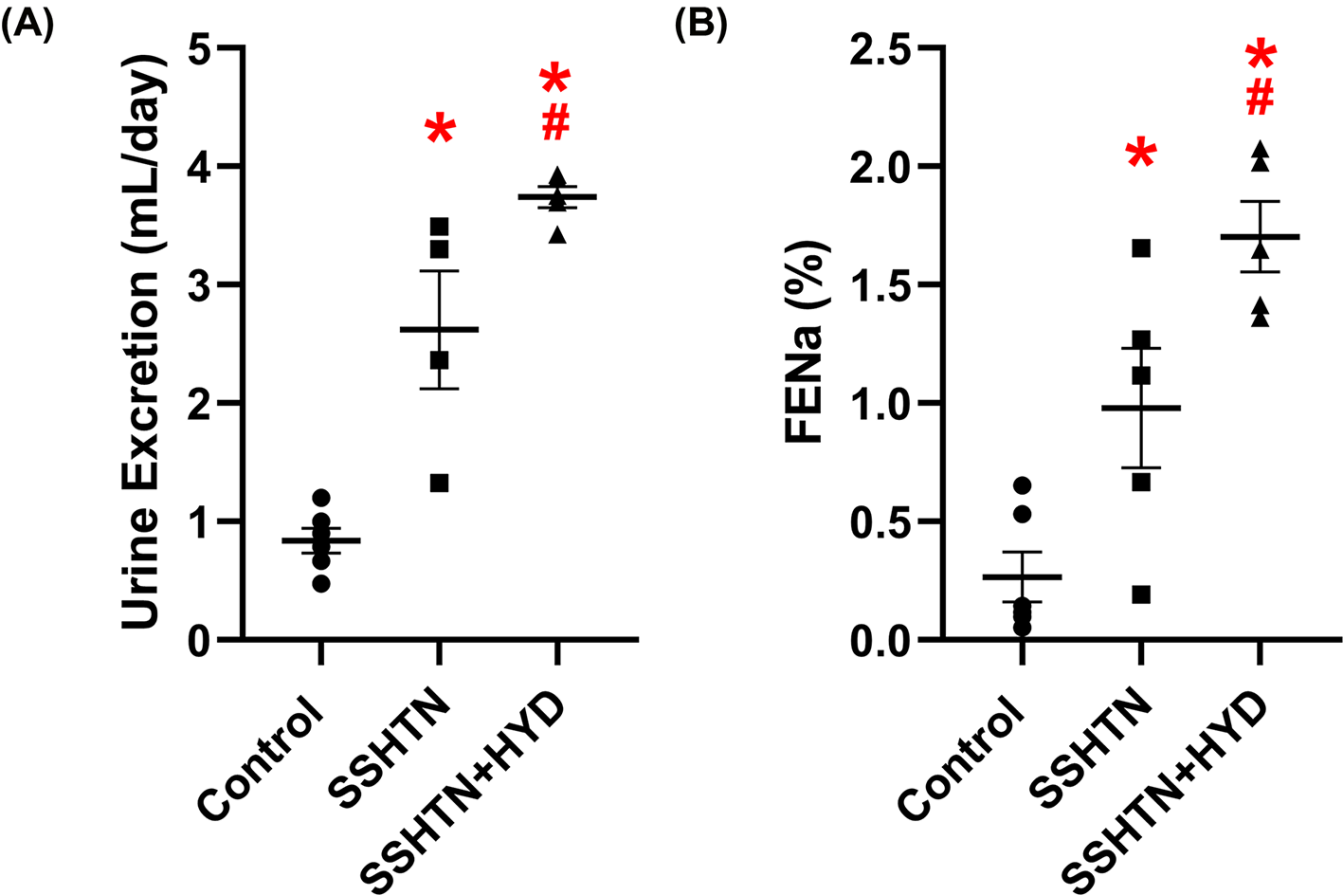
Hydralazine treatment improved kidney function in male mice with SSHTN (**A**) 24-h urine excretion and (**B**) fractional excretion of sodium (FENa) in control, SSHTN, and SSHTN+HYD male mice. Results are presented as mean ± SEM and all statistical analyses were performed with one-way ANOVA (*n* = 4−6 per group). *P<0.05 vs control mice and #*P*<0.05 vs SSHTN mice.

Next, we assessed the impact of BP reduction on renal function in male SSHTN mice by collecting and analyzing urine. We observed a notable increase in urine output in both SSHTN and SSHTN+HYD mice compared with the control group (Figure 4A). Additionally, HYD treatment significantly increased urine output in SSHTN+HYD mice compared with SSHTN mice (Figure 4A). Similarly, an increase in FENa was observed in both SSHTN and SSHTN+HYD mice compared with the control group, and the SSHTN+HYD group also had a further increase in FENa relative to the SSHTN group (Figure 4B).

### HYD treatment improved testicular immune cells and reduced testicular inflammation in male SSHTN mice

**Figure 5:**
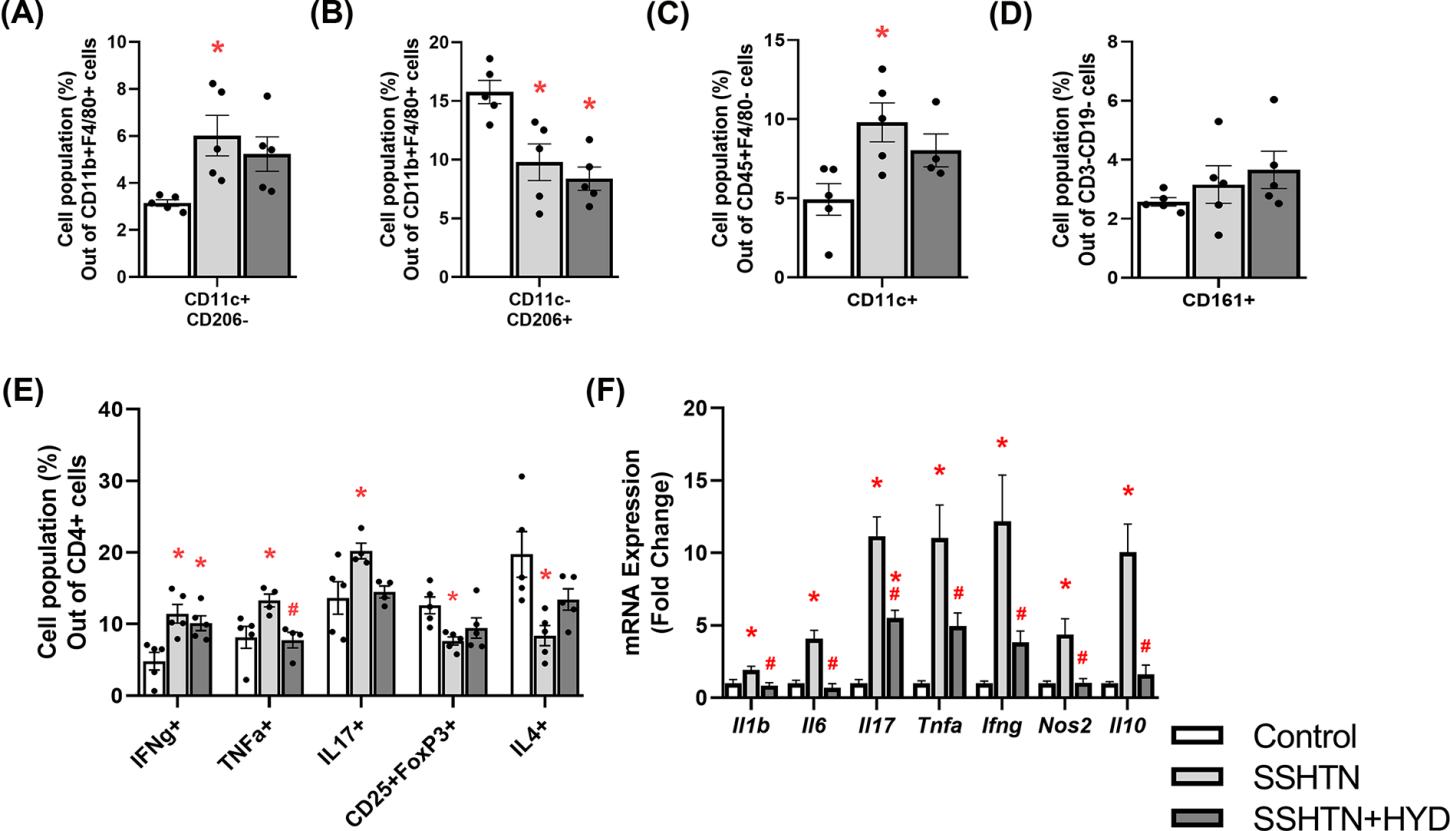
Hydralazine treatment favorably altered immune cells and decreased inflammation in the testes of male mice with SSHTN Flow cytometric data examining testicular populations of (**A**) M1 macrophages, (**B**) M2 macrophages, (**C**) DCs, (**D**) NK cells, and (**E**) T cells in control, SSHTN, and SSHTN+HYD male mice. Each cell population is shown as a percentage of its respective parent population. (**F**) Testicular cytokine expression changes in control, SSHTN, and SSHTN+HYD male mice. Results are presented as mean ± SEM and statistical analyses were performed with one-way ANOVA (*n* = 4−6 per group). *P<0.05 vs control mice and #*P*<0.05 vs SSHTN mice.

We reported that SSHTN induces elevated pro-inflammatory macrophages while concurrently diminishing anti-inflammatory macrophages in the testes [7]. To investigate whether the reduction of BP impacts testicular immune cells, we performed flow cytometry. Testicular M1 macrophages were increased significantly in SSHTN mice when compared with control mice but were not significantly altered in SSHTN+HYD mice compared with controls (Figure 5A). There was a significant decrease in M2 macrophages within the testes of SSHTN and SSHTN+HYD mice when compared with testes from the control group (Figure 5B). DCs were increased significantly in the testes of SSHTN mice compared with those of control mice, while no significant change was noted in the testes of SSHTN+HYD mice compared with controls (Figure 5C). There were no observed alterations in NK cell populations in the testes of SSHTN or SSHTN+HYD mice (Figure 5D). Testicular CD4+IFNg+ Th1 cells were increased in SSHTN and SSHTN+HYD mice when compared with control mice (Figure 5E). Significant increases in CD4+TNFa+ Th1 cells and Th17 cells were observed in the testes of SSHTN mice when compared with those of control mice (Figure 5E). In SSHTN+HYD mice, HYD treatment resulted in a significant decrease in testicular CD4+TNFa+ Th1 cells and a trend towards a decrease in testicular Th17 cells when compared with SSHTN mice (Figure 5E). Testicular Tregs and Th2 cells were notably decreased in SSHTN mice compared with control mice, while these populations were unchanged in SSHTN+HYD mice compared with controls (Figure 5E).

To delve deeper into the impact of lowering BP on the scope of testicular inflammation, we analyzed cytokine gene expression. The testes of SSHTN mice exhibited a significant increase in the expression of *Il1b, Il6, Tnfa, Ifng, Nos2*, and *Il10* compared with those of controls, while the testes of SSHTN+HYD mice demonstrated a decrease in the expression of these genes relative to SSHTN testes (Figure 5F). There was a significant increase in *Il17* expression in the testes of both SSHTN and SSHTN+HYD mice compared with those of control mice (Figure 5F). However, a significant decrease in testicular *Il17* expression was observed in SSHTN+HYD mice when compared with SSHTN mice (Figure 5F).

### HYD treatment did not alleviate testicular lymphangiogenesis in male SSHTN mice

**Figure 6:**
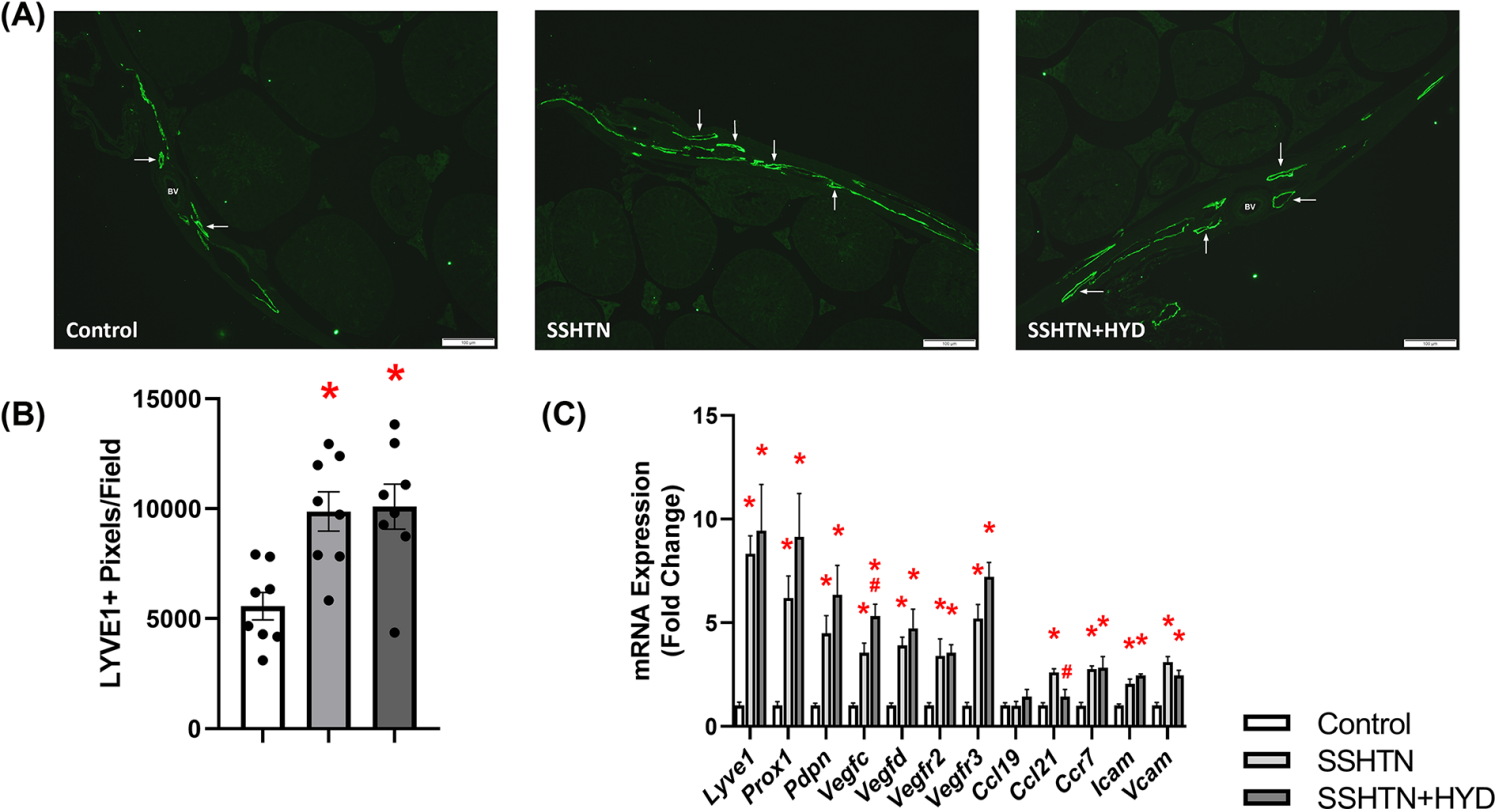
Hydralazine treatment did not alter lymphatics in the testes of male mice with SSHTN (**A**) LYVE1 immunofluorescence in the testes of control, SSHTN, and SSHTN+HYD male mice. LYVE1 is labelled green. Lymphatic vessels are indicated with arrows. Images were taken in the outer tunica albuginea at 10×. Scale bars = 100 µm. (**B**) Testicular lymphatic density in control, SSHTN, and SSHTN+HYD male mice as determined by quantification of LYVE1+ pixels per field (*n* = 8 per group). (**C**) Testicular expression changes in lymphangiogenesis-related genes in control, SSHTN, and SSHTN+HYD male mice (*n* = 6 per group). Results are presented as mean ± SEM and all statistical analyses were performed with one-way ANOVA. *P<0.05 vs control mice and #*P*<0.05 vs SSHTN mice.

To explore the impact of lowering BP on testicular lymphatics, tissue sections underwent immunofluorescent staining and imaging. Quantification of LYVE1+ pixels revealed a substantial increase in lymphatic vessel density in the testes of both SSHTN and SSHTN+HYD mice compared with the testes of control mice (Figure 6A,B). Furthermore, both the SSHTN and SSHTN+HYD groups had elevated testicular expression of the lymphatic vessel markers *Lyve1, Prox1*, and *Pdpn*, lymphangiogenic growth factors *Vegfc* and *Vegfd*, vascular endothelial growth factor receptors *Vegfr2* and *Vegfr3*, and adhesion molecules *Icam* and *Vcam* (Figure 6C). HYD treatment further increased *Vegfc* expression in SSHTN+HYD mice compared with SSHTN mice (Figure 6C). Additionally, SSHTN and SSHTN+HYD mice had increased testicular expression of the chemokine receptor *Ccr7* compared with control mice (Figure 6C). Testicular expression of the lymphatic-specific chemokine *Ccl21* was increased in SSHTN mice compared with controls and significantly decreased in SSHTN+HYD mice compared with SSHTN mice (Figure 6C). Collectively, these results suggest a persistent elevation in testicular lymphatic density among both SSHTN groups regardless of HYD treatment, suggesting an intricate link between inflammation and lymphatic regulation that may not be fully alleviated by BP reduction alone.

### HYD treatment improved testicular function in male SSHTN mice

**Figure 7:**
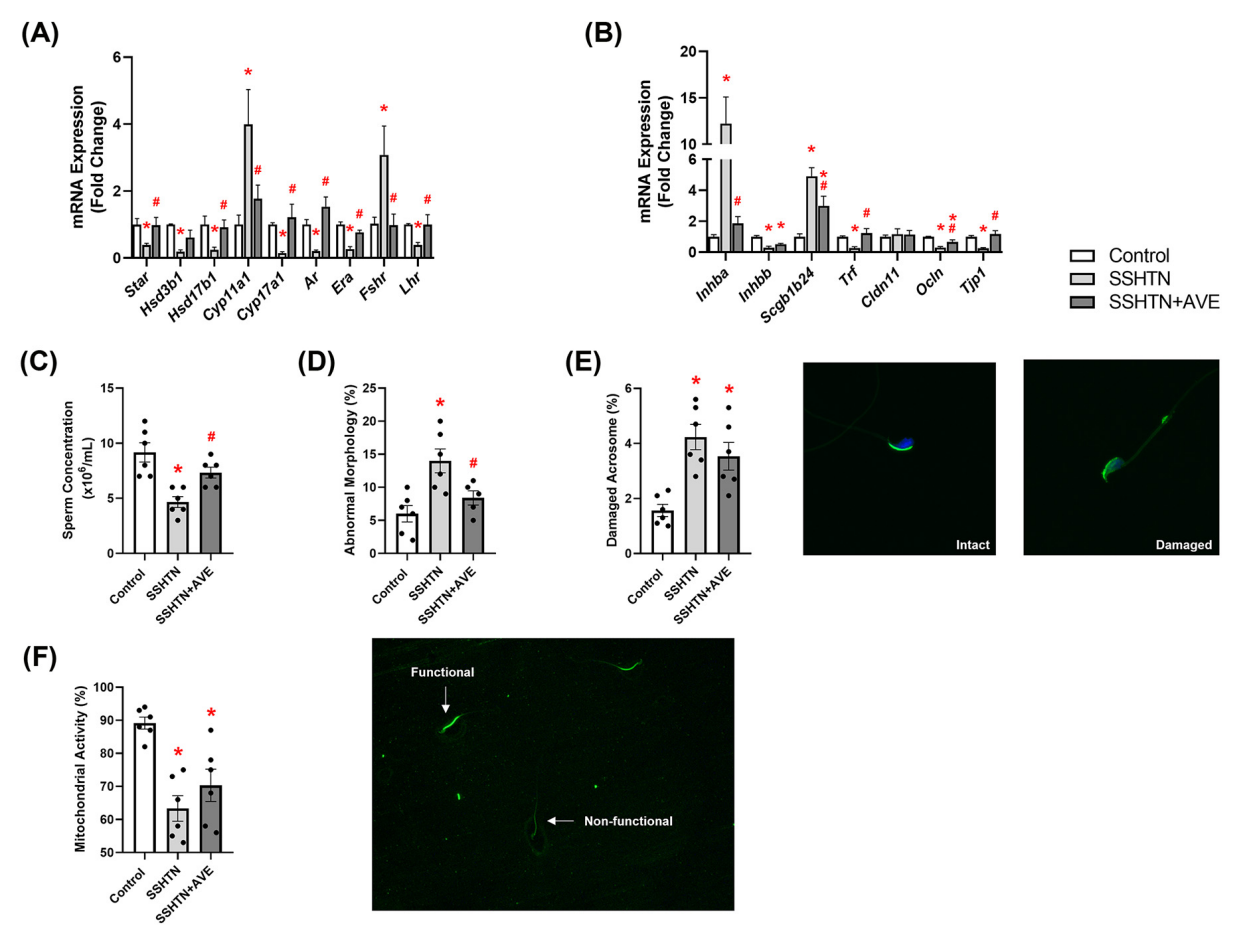
Hydralazine treatment improved testicular function and improved sperm health in male mice with SSHTN Testicular expression changes in (**A**) steroidogenic pathway-related genes and hormone receptors and (**B**) secretory and tight junction proteins in control, SSHTN, and SSHTN+HYD male mice (*n* = 5−6 per group). Measures of sperm (**C**) concentrations, (**D**) morphology, (**E**) mitochondrial activity levels, and (**F**) acrosome integrity to assess sperm quality and reproductive health in control, SSHTN, and SSHTN+HYD male mice (*n* = 6−8 per group). Results are presented as mean ± SEM and statistical analyses were performed with one-way ANOVA. *P<0.05 vs control mice and #*P*<0.05 vs SSHTN mice.

To gain a comprehensive understanding of the impact of lowered BP on testicular function, we analyzed mRNA expression levels of steroidogenic pathway genes, hormone receptors, and secretory and tight junction proteins by qRT-PCR. There was a significant decrease in the expression of *Star*, which regulates cholesterol transport across the mitochondrial membrane, in the testes of SSHTN mice compared with control testes (Figure 7A). HYD treatment prevented this change in SSHTN+HYD mice, which showed testicular *Star* expression levels comparable to those of control mice (Figure 7A). mRNA expression of *Hsd3b1* was decreased in the testes of both SSHTN and SSHTN+HYD mice when compared with control testes, while there was no change in the expression of *Cyp11a1* (Figure 7A). There was a significant decrease in testicular expression of *Hsd17b1* and *Cyp17a1* in SSHTN mice compared with control mice, whereas HYD treatment significantly increased testicular expression of the same genes when compared with SSHTN mice (Figure 7A). A significant decrease in *Ar* expression was observed in the testes of SSHTN and SSHTN+HYD mice compared with control testes (Figure 7A). SSHTN testes had a significant decrease in *Era* and *Lhr* expression (Figure 7A). However, SSHTN+HYD testes exhibited a significant increase in the expression of *Era* in reference to SSHTN testes but showed no change in *Lhr* expression (Figure 7A). In the testes of SSHTN mice, there was an increase in *Inhba* and *Scgb1b24* expression when compared with control testes, and this was rescued in the SSHTN+HYD testes (Figure 7B). Additionally, there was a significant decrease in testicular expression of *Inhbb* and *Trf* in SSHTN mice when compared with control mice (Figure 7B). However, when compared with the SSHTN group, SSHTN+HYD mice had an increase in testicular expression of these genes (Figure 7B). Further, we observed that *Ocln* was decreased in the testes of both SSHTN and SSHTN+HYD mice compared with the testes of control mice, whereas *Cldn11* expression remained unchanged in both hypertensive groups (Figure 7B). The testes of SSHTN mice exhibited a significant decrease in the expression of *Tjp1* when compared with control testes (Figure 7B). *Tjp1* expression was increased in SSHTN+HYD testes compared with SSHTN testes (Figure 7B). These data indicate that HYD treatment was protective against many gene expression changes associated with testicular dysfunction.

To investigate potential BP-related improvements in gonadal dysfunction associated with SSHTN, we conducted a comprehensive analysis of various sperm parameters. This included the assessment of sperm concentration and morphology, as well as examinations of acrosome integrity and mitochondrial activity. A decrease in sperm concentration was observed in both SSHTN and SSHTN+HYD mice compared with control mice (Figure 7C). However, HYD treatment led to a significant increase in sperm concentration in SSHTN+HYD mice compared with SSHTN mice (Figure 7C). A significant increase in the percentage of sperm with abnormal morphology was evident in SSHTN mice, while SSHTN+HYD mice showed a significant decrease in abnormal sperm compared with SSHTN mice (Figure 7D). There was a significant decrease in the percentage of sperm with functional mitochondria in both SSHTN and SSHTN+HYD mice when compared with control mice (Figure 7E). There was a significant increase in the percentage of sperm with damaged acrosomes in SSHTN mice compared with control mice (Figure 7F). HYD treatment decreased the percentage of dysfunctional sperm in the SSHTN+HYD mice compared with SSHTN mice (Figure 7F). Overall, these results offer illumination into the nuanced connection between BP and testicular function.

### HYD treatment attenuated BP, renal pro-inflammatory immune cells, and renal inflammation in female SSHTN mice

**Figure 8:**
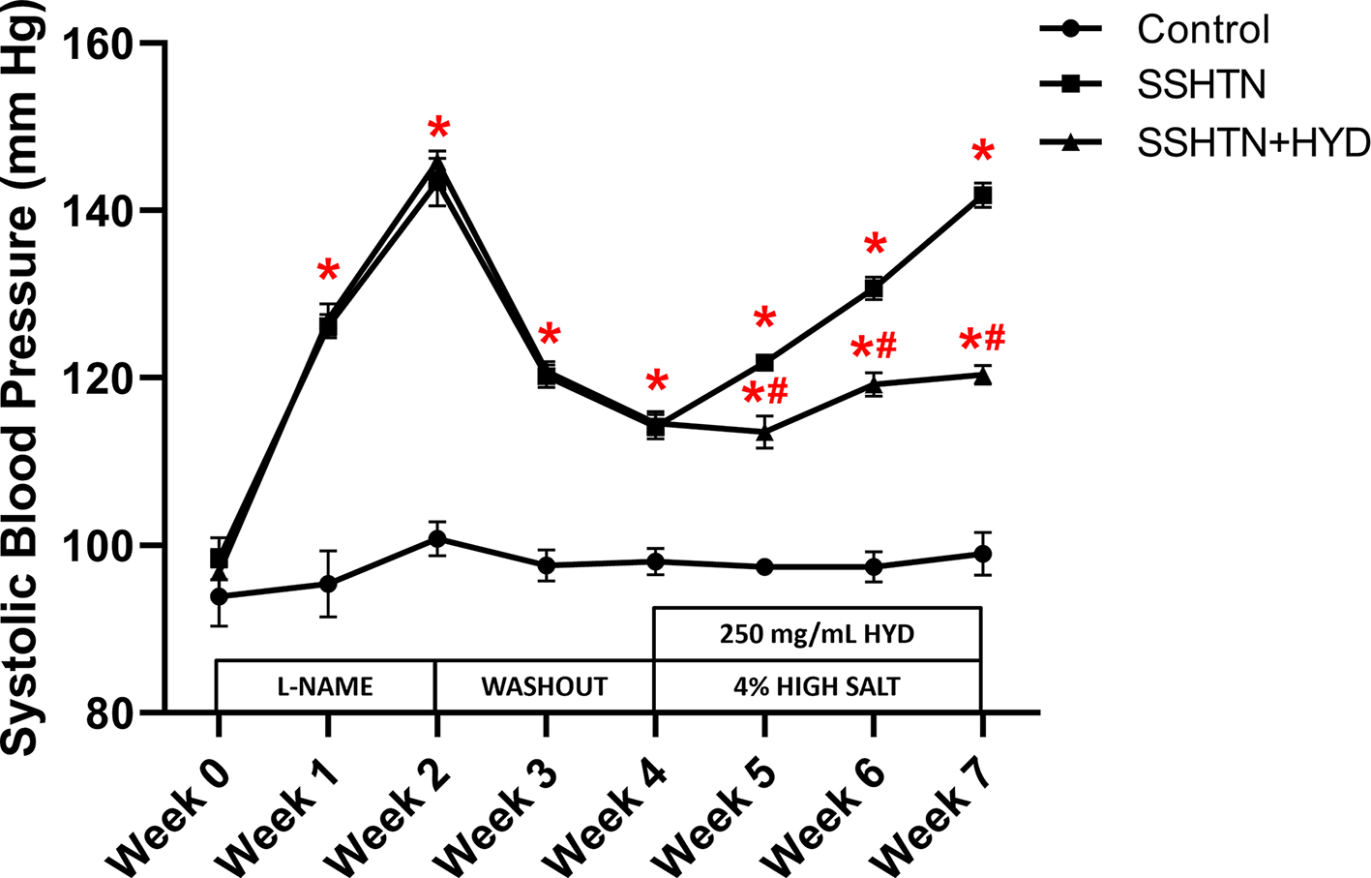
Hydralazine treatment attenuated SBP in female mice with SSHTN SBP measurements in untreated control, SSHTN, and SSHTN+HYD female mice. Blood pressures were taken weekly via tail cuff. Results are presented as mean ± SEM and statistical analyses were performed with one-way ANOVA (*n* = 5 per group). *P<0.05 vs control mice and #*P*<0.05 vs SSHTN mice.

We conducted a parallel study with female mice to investigate whether the reduction of BP has sex-specific effects on pro-inflammatory immune cells and inflammation in the kidneys of SSHTN mice. Both female hypertensive groups exhibited increased SBP compared with control mice throughout the duration of the model (Figure 8). The rise in BP was effectively attenuated with HYD treatment, as the female SSHTN+HYD mice had significantly decreased SBP compared with the female SSHTN mice at all weeks of the high salt diet phase (Figure 8).

**Figure 9:**
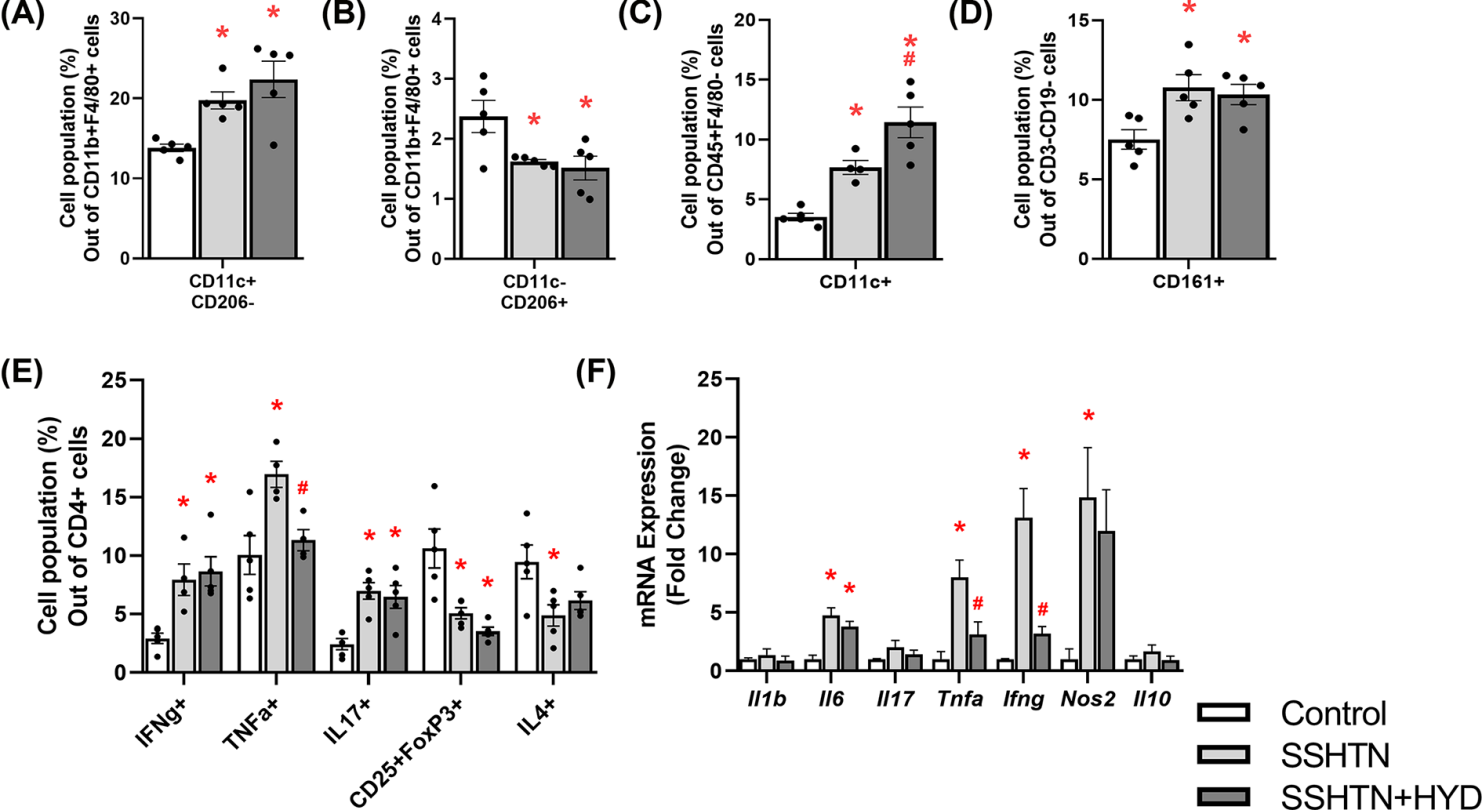
Hydralazine treatment differentially altered immune cells and inflammation in the kidneys of female mice with SSHTN Flow cytometric data examining renal populations of (**A**) M1 macrophages, (**B**) M2 macrophages, (**C**) DCs, (**D**) NK cells, and (**E**) T cells in control, SSHTN, and SSHTN+HYD female mice (*n* = 4-5 per group). Each cell population is shown as a percentage of its respective parent population. (**F**) Renal cytokine expression changes in control, SSHTN, and SSHTN+HYD female mice (*n* = 3-6 per group). Results are presented as mean ± SEM and statistical analyses were performed with one-way ANOVA. *P<0.05 vs control mice and #*P*<0.05 vs SSHTN mice.

Single cell suspensions prepared from kidneys were immunophenotyped by flow cytometry. Surprisingly, the data revealed a significant increase in M1 macrophages (Figure 9A) along with a decrease in M2 macrophages in the kidneys of both female SSHTN and SSHTN+HYD mice (Figure 9B). SSHTN and SSHTN+HYD kidneys had increased DCs compared with control kidneys (Figure 9C). SSHTN+HYD mice exhibited a significant increase in renal DCs compared with SSHTN mice (Figure 9C). A notable increase in renal NK cells was also observed in both SSHTN and SSHTN+HYD mice when compared with control mice (Figure 9D). There were significant increases in CD4+IFNg+ Th1 cells and Th17 cells in the kidneys of both SSHTN and SSHTN+HYD mice compared with those of control mice (Figure 9E). Conversely, CD4+TNFa+ Th1 cells were increased in the kidneys of SSHTN mice compared with kidneys of control mice, and this increase was mitigated in SSHTN+HYD mice when compared with SSHTN mice (Figure 9E). On the other hand, renal Tregs decreased significantly in both SSHTN and SSHTN+HYD mice compared with control mice (Figure 9E). Additionally, SSHTN kidneys demonstrated a significant decrease in Th2 cells compared with control kidneys; however, the SSHTN+HYD group did not experience this change (Figure 9E).

Our subsequent emphasis was on unraveling the complex interplay between BP reduction and inflammatory markers within renal tissues of female SSHTN mice. Analysis of qRT-PCR data unveiled an increase in *Il6* levels in the kidneys of both SSHTN and SSHTN+HYD mice when compared with control kidneys, while *Il1b, Il17*, and *Il10* exhibited no significant alterations (Figure 9F). The expression levels of *Tnfa, Ifng*, and *Nos2* were increased significantly in the kidneys of SSHTN mice compared with the kidneys of control mice, while SSHTN+HYD mice exhibited significant decreases in renal *Tnfa* and *Ifng* expression when compared with SSHTN mice (Figure 9F).

### HYD treatment decreased inflammation-associated renal lymphangiogenesis in female SSHTN mice

**Figure 10:**
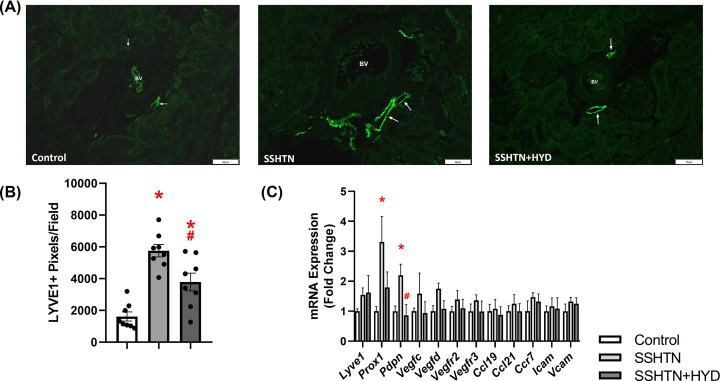
Hydralazine treatment decreased lymphatics in the kidneys of female mice with SSHTN (**A**) LYVE1 immunofluorescence in the kidneys of control, SSHTN, and SSHTN+HYD female mice. LYVE1 is labelled green and lymphatic vessels are indicated with arrows surrounding the blood vessels (BV). Images were taken in the cortex at 20×. Scale bars = 50 µm. (**B**) Renal lymphatic density in control, SSHTN, and SSHTN+HYD female mice as determined by quantification of LYVE1+ pixels per field (*n* = 8 per group). (**C**) Renal expression changes in lymphangiogenesis-related genes in control, SSHTN, and SSHTN+HYD female mice (*n* = 3–5 per group). Results are presented as mean ± SEM and all statistical analyses were performed with one-way ANOVA. *P<0.05 vs control mice and #*P*<0.05 vs SSHTN mice.

Continuing our investigation in females, we analyzed fluorescent-labelled kidney sections to determine potential sex-specific differences in renal lymphatic density in response to BP lowering. A significant increase in renal lymphatic density was observed in female SSHTN and SSHTN+HYD mice compared with control mice; however, SSHTN+HYD mice had decreased lymphatic density compared with SSHTN mice (Figure 10A,B). Gene expression studies revealed significant increases of *Prox1* and *Pdpn* in the kidneys of SSHTN mice when compared with control kidneys (Figure 10C). Notably, there was a significant decrease of *Pdpn* in SSHTN+HYD mice compared with SSHTN mice, while no changes were observed in the expression of other lymphatic vessel markers, chemokines, and their receptors (Figure 10C).

### HYD treatment did not improve renal function in female SSHTN mice

**Figure 11:**
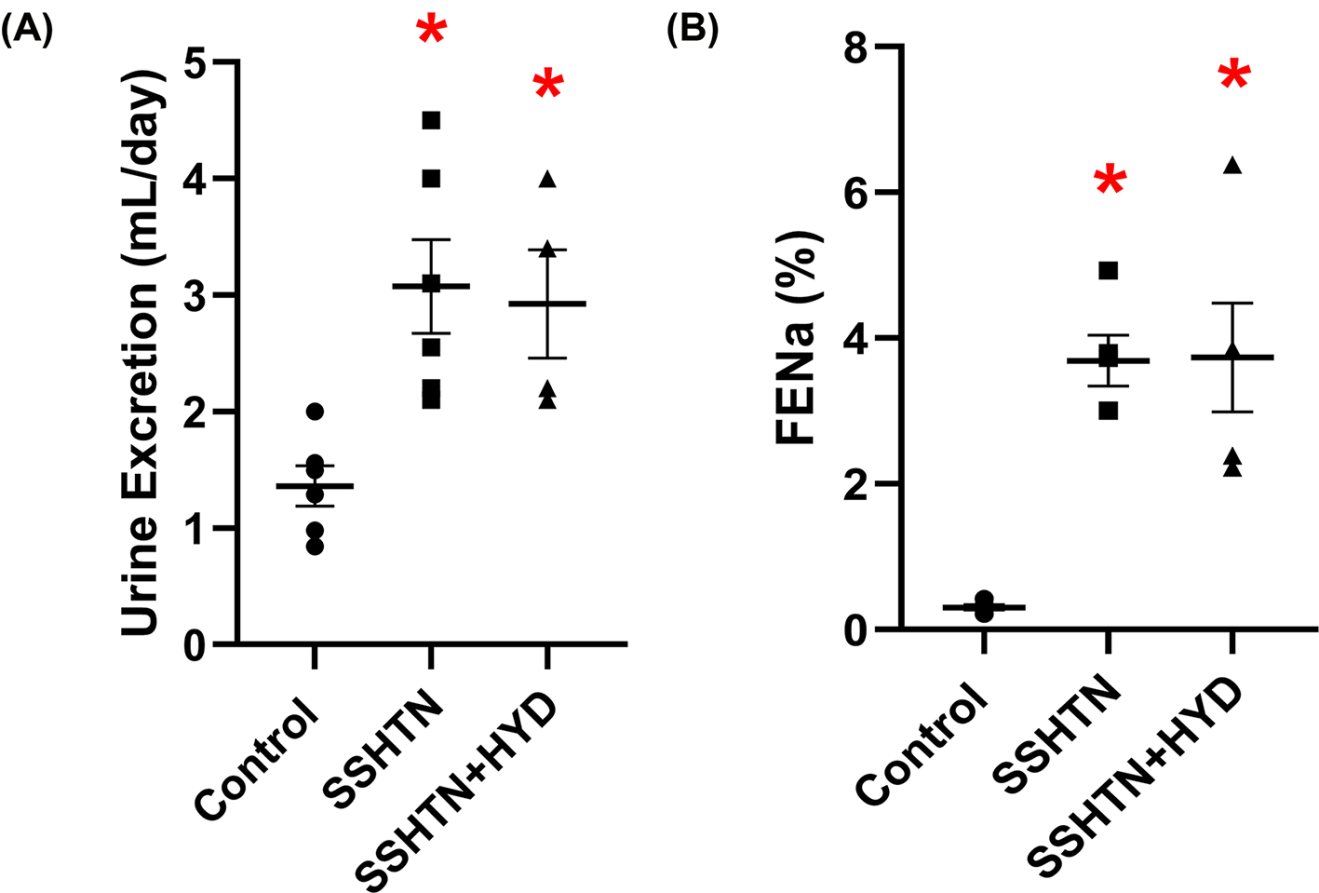
Hydralazine treatment did not improve kidney function in female mice with SSHTN (**A**) 24-h urine excretion and (**B**) fractional excretion of sodium (FENa) in control, SSHTN, and SSHTN+HYD female mice. Results are presented as mean ± SEM and all statistical analyses were performed with one-way ANOVA s (*n* = 4–6 per group). *P<0.05 vs control mice and #*P*<0.05 vs SSHTN mice.

Next, we aimed to assess potential improvements in renal function following BP reduction. There was a significant increase in urine output in both female SSHTN and SSHTN+HYD mice compared with control mice (Figure 11A). Similarly, a significant increase in FENa was observed in both SSHTN and SSHTN+HYD mice compared with control mice (Figure 11B).

### HYD treatment differentially affected ovarian immune cells and inflammation in female SSHTN mice

**Figure 12:**
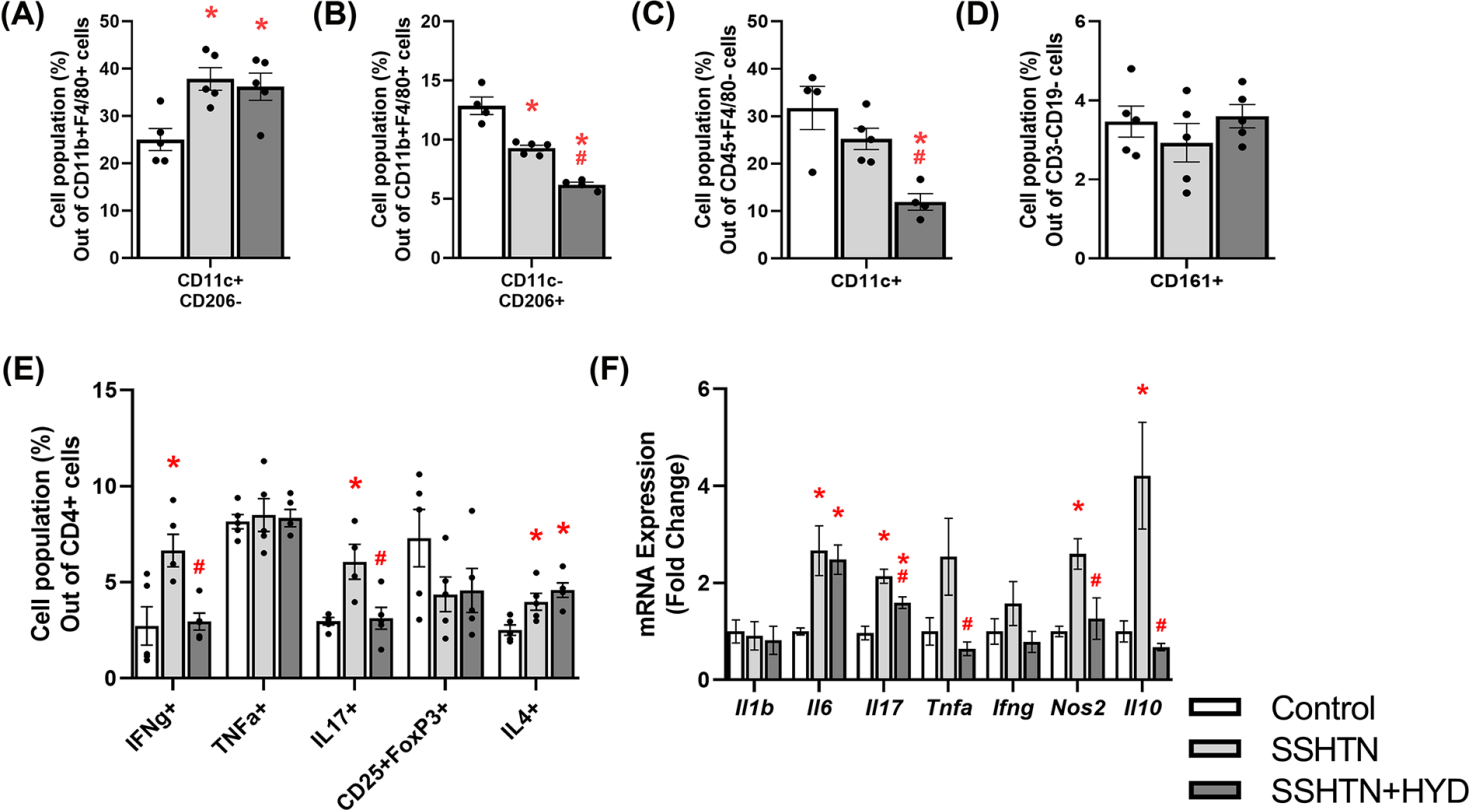
Hydralazine treatment differentially affected immune cells and inflammation in the ovaries of female mice with SSHTN Flow cytometric data examining ovarian populations of (**A**) M1 macrophages, (**B**) M2 macrophages, (**C**) DCs, (**D**) NK cells, and (**E**) T cells in control, SSHTN, and SSHTN+HYD female mice (*n* = 4–5 per group). Each cell population is shown as a percentage of its respective parent population. (**F**) Ovarian cytokine expression changes in control, SSHTN, and SSHTN+HYD female mice (*n* = 5–6 per group). Results are presented as mean ± SEM and statistical analyses were performed with one-way ANOVA. *P<0.05 vs control mice and #*P*<0.05 vs SSHTN mice.

Immunophenotyping of ovarian cells by flow cytometry revealed increased M1 macrophages (Figure 12A) and decreased M2 macrophages in SSHTN and SSHTN+HYD ovaries compared with control ovaries (Figure 12B). Interestingly, HYD treatment further decreased ovarian M2 macrophages in SSHTN+HYD mice when compared with SSHTN mice (Figure 12B). Ovarian DCs were decreased in the SSHTN+HYD group compared with both the control and SSHTN groups, whereas the SSHTN group did not show any changes compared with controls (Figure 12C). Ovarian NK cells exhibited no discernible changes in response to the experimental conditions (Figure 12D). Ovarian CD4+IFNg+ Th1 and Th17 cells were increased in SSHTN mice compared with control mice (Figure 12E). However, the SSHTN+HYD group had significantly decreased CD4+IFNg+ Th1 and Th17 cells compared with the SSHTN group (Figure 12E). There was a significant increase in Th2 cells in the ovaries of SSHTN and SSHTN+HYD mice when compared with control ovaries (Figure 12E). Meanwhile, neither CD4+TNFa+ Th1 cells nor Tregs were significantly affected by either of the experimental conditions (Figure 12E).

Cytokine gene expression analysis revealed a significant increase in *Il6* and *Il17* expression in the ovaries of both SSHTN and SSHTN+HYD mice compared with the ovaries of control mice (Figure 12F). HYD treatment resulted in a significant decrease in ovarian *Il17* expression in the SSHTN+HYD group when compared with the SSHTN group (Figure 12F). Ovaries from the SSHTN+HYD group exhibited a significant decrease in *Tnfa* expression when compared with SSHTN ovaries (Figure 12F). Ovarian *Il1b* and *Ifng* expression remained unchanged in both groups (Figure 12F). Expression levels of *Nos2* and *Il10* were increased significantly in the ovaries of SSHTN mice compared with control ovaries, while they were decreased significantly in the SSHTN+HYD group compared with the SSHTN group (Figure 12F).

### HYD partially alleviated increased ovarian lymphatic density in female SSHTN mice

**Figure 13:**
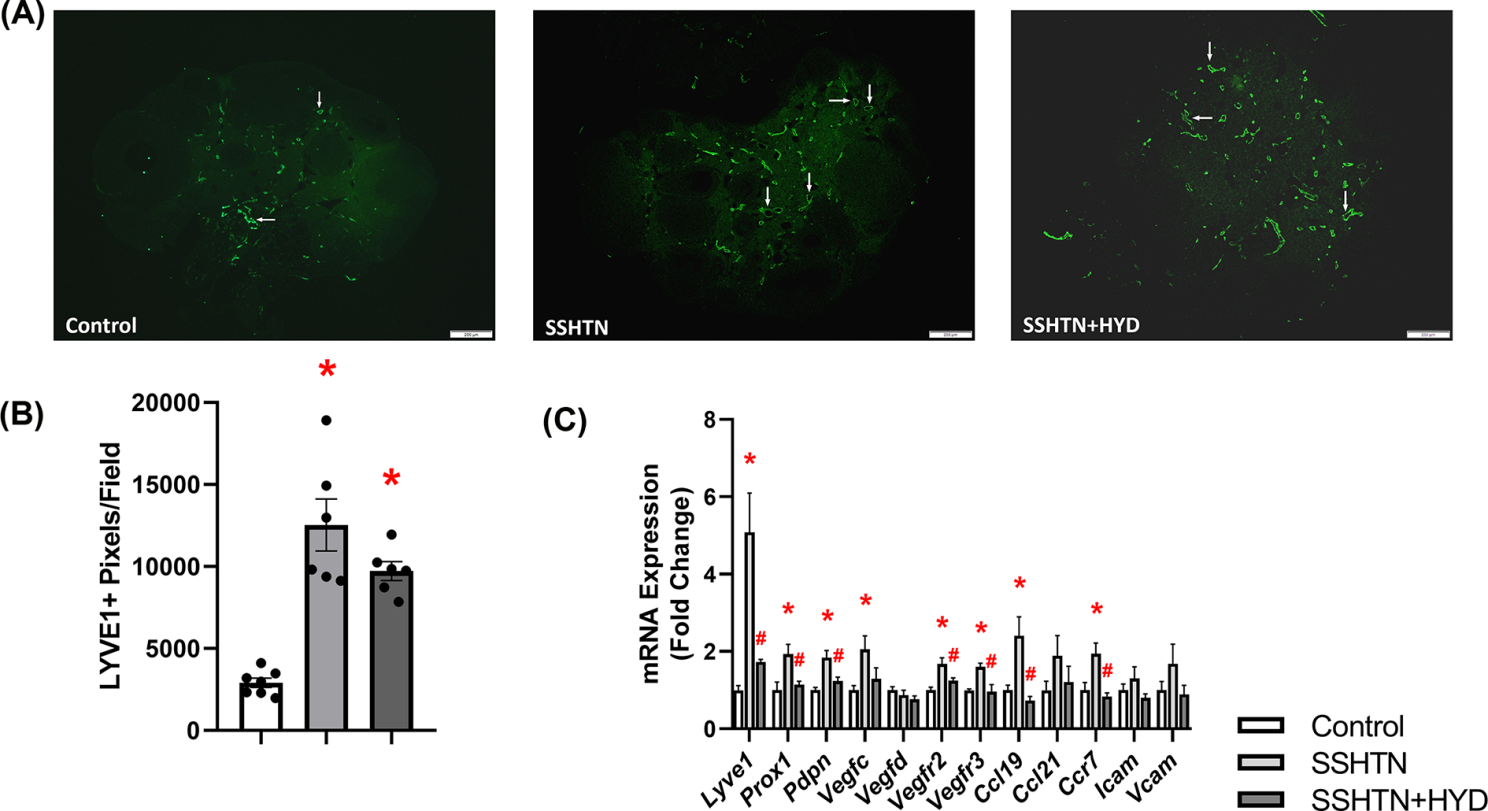
Hydralazine treatment partially decreased lymphangiogenesis in the ovaries of female mice with SSHTN (**A**) LYVE1 immunofluorescence in the ovaries of control, SSHTN, and SSHTN+HYD female mice. LYVE1 is labelled green. Lymphatic vessels are indicated with arrows. Images were taken of the whole ovary at 4x. Scale bars = 200µm. (**B**) Ovarian lymphatic density in control, SSHTN, and SSHTN+HYD female mice as determined by quantification of LYVE1+ pixels per field (*n* = 8 per group). (**C**) Ovarian expression changes in lymphangiogenesis-related genes in control, SSHTN, and SSHTN+HYD female mice (*n* = 5-6 per group). Results are presented as mean ± SEM and all statistical analyses were performed with one-way ANOVA. *P<0.05 vs control mice and #*P*<0.05 vs SSHTN mice.

We utilized immunofluorescence to assess the influence of BP reduction on ovarian lymphatic density, aiming to discern potential sex-specific differences. Our findings observed a significant increase in lymphatic vessel density in the ovaries of both SSHTN and SSHTN+HYD mice when compared with ovaries from control mice (Figure 13A,B). Ovaries of SSHTN mice exhibited significant increases in the expression of *Lyve1, Prox1, Pdpn, Vegfc, Vegfr2, Vegfr3, Ccl19*, and *Ccr7* compared with control ovaries (Figure 13C). HYD treatment alleviated the increase in expression of these genes, with the exception of *Vegfc*, in the SSHTN+HYD group when compared with the SSHTN group (Figure 13C). These findings suggest that, although BP reduction somewhat attenuated pro-inflammatory immune cells and inflammation in the ovaries of SSHTN mice, it was not sufficient to completely alleviate inflammation-associated lymphangiogenesis.

### HYD partially restored ovarian function in female SSHTN mice

**Figure 14:**
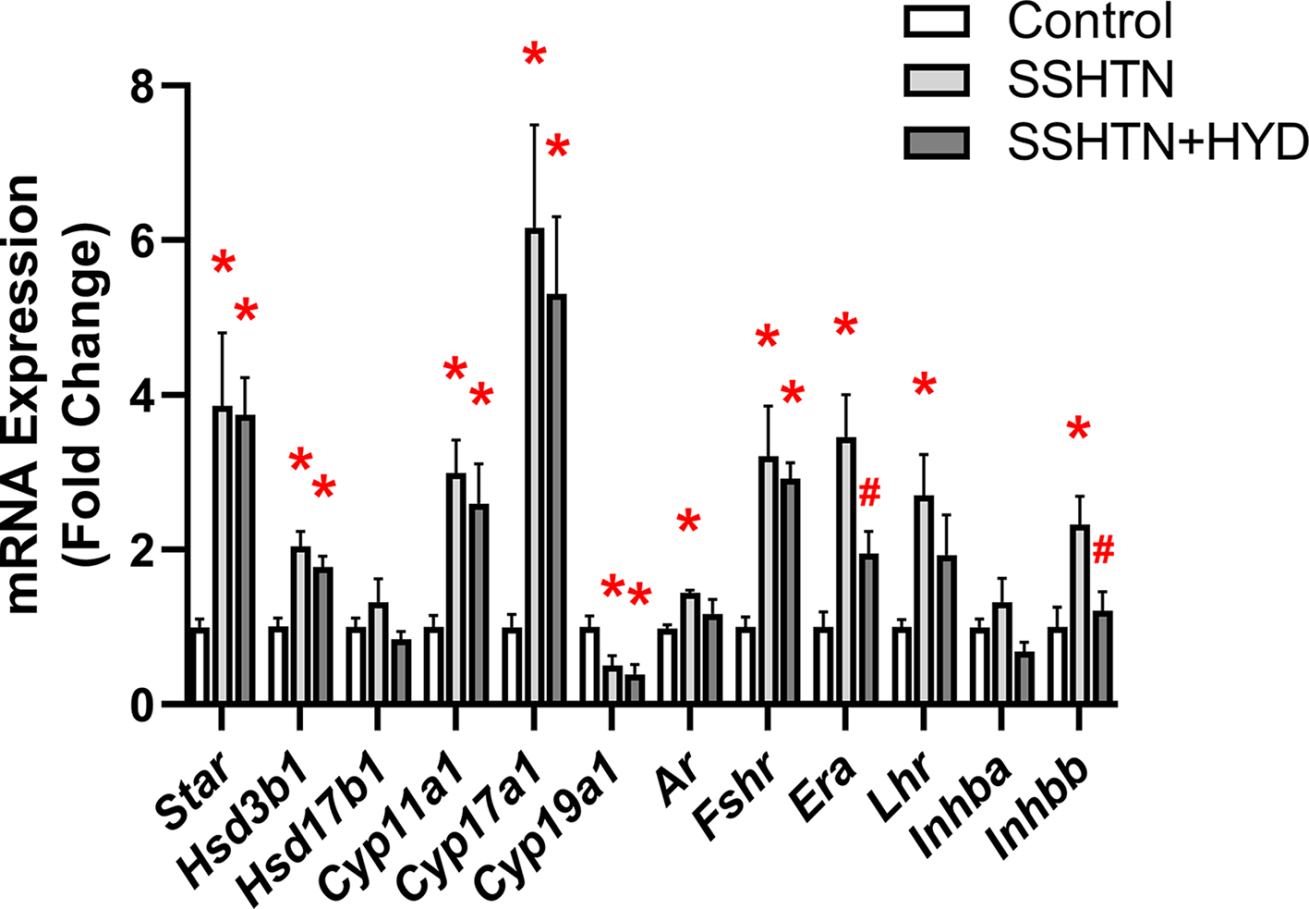
Hydralazine treatment partially improved ovarian function in female mice with SSHTN Ovarian expression changes in cholestrol transport proteins, steroidogenic enzymes, and hormone receptors in control, SSHTN, and SSHTN+HYD female mice. Results are presented as mean ± SEM and statistical analyses were performed with one-way ANOVA (*n* = 5–6 per group). *P<0.05 vs control mice and #*P*<0.05 vs SSHTN mice.

Subsequently, we sought to examine potential improvements in ovarian function associated with BP reduction by assessing gene expression levels of cholesterol transport proteins, steroidogenic enzymes, and hormone receptors. In the ovaries of both SSHTN and SSHTN+HYD mice, we noted a significant increase in the gene expression of *Star, Hsd3b1, Cyp11a1*, and *Cyp17a1* compared with control ovaries, while *Hsd17b1* expression remained unchanged in all groups (Figure 14). Conversely, there was a decrease in the expression of *Cyp19a1*, the aromatase enzyme that converts androgen precursors to estrogen, in both SSHTN and SSHTN+HYD ovaries compared with control ovaries (Figure 14). The ovaries of SSHTN mice demonstrated a significant increase in the expression of hormone receptors *Ar, Era*, and *Lhr* when compared with control ovaries, and *Era* expression was decreased in SSHTN+HYD ovaries compared with SSHTN ovaries (Figure 14). Moreover, ovarian *Fshr* expression was increased in both SSHTN and SSHTN+HYD mice compared with control mice (Figure 14). Expression of *Inhbb* was significantly increased in the ovaries of SSHTN mice compared with those of control mice and significantly decreased in SSHTN+HYD ovaries compared with SSHTN ovaries (Figure 14). *Inhba* expression remained unchanged in ovaries from both experimental groups (Figure 14). These findings demonstrate that BP reduction was not sufficient to fully restore ovarian function, underscoring the complexity of the relationship between BP regulation and ovarian health in SSHTN and highlighting the need for further exploration of contributing factors and mechanisms.

## Discussion

Investigations from our lab and others have highlighted the association between SSHTN and increased pro-inflammatory immune cells, inflammation, and inflammation-associated lymphangiogenesis, leading to end organ damage in both renal and gonadal tissues of mice [7,22,23,26,30,31]. The ambiguity regarding whether these detrimental effects primarily stem from increased systemic pressure, elevated salt levels, or a combination of both led us to hypothesize that lowering BP alone would not suffice to alleviate the renal and gonadal inflammation and end-organ damage linked to SSHTN. To address this question, our current study employed a mouse model of SSHTN and administered HYD, a BP lowering agent, to discern the effects of BP reduction on immune cell populations, inflammation, and lymphatics in the kidneys and gonads of mice. Additionally, this investigation explored potential sex-specific differences in response to HYD by including both male and female mice. Our findings indicate that, in addition to lowering BP, HYD administration differentially affected pro-inflammatory immune cells, inflammation, and lymphatic density in the kidneys and gonads of both male and female mice with SSHTN.

Pharmacological reduction of BP, facilitated by HYD in the current study, mostly improved various parameters associated with the immune response, inflammation, lymphatics, and organ function. Notably, in male SSHTN mice, decreasing BP attenuated pro-inflammatory immune cells, such as DCs in the kidney and CD4+ IFNg+ Th1 cells in the testes. In females, notably attenuated pro-inflammatory immune cell populations included CD4+ TNFa+ Th1 cells in the kidney and CD4+ IFNg+ Th1 cells and Th17 cells in the ovaries. These results highlight the direct impact of BP on several distinct immune cell populations, contributing to our understanding of the complex relationship between BP and immune cells influenced by sex-specific factors and hormones. Future studies exploring the intricate cross-talk between sex hormones, salt, and the immune system in SSHTN could provide further insight into the mechanisms underlying these observed differences and pave the way for tailored therapeutic strategies.

Interestingly, the reduction in BP demonstrated a consistent and notable impact on mitigating inflammation in both renal and gonadal tissues across both sexes. This observation suggests a shared sensitivity of renal and gonadal tissues to BP levels and underlines the broad anti-inflammatory effects of HYD, suggesting potential systemic benefits in SSHTN. HYD, renowned for its efficacy as a vasodilator in HTN, has recently emerged as a potential anti-inflammatory agent, expanding its therapeutic implications [34–37]. The multifaceted nature of HYD adds another layer of complexity to our understanding. While our study demonstrates an association of improved renal and gonadal inflammation with lowered BP, teasing apart the specific anti-inflammatory mechanisms of HYD remains a challenge. This poses a limitation, emphasizing the need for dedicated and targeted investigations to unravel the specific impact of HYD on diverse immune cell populations.

SSHTN has been extensively linked to inflammation and end organ damage, establishing a significant association between high salt intake and the development and exacerbation of HTN [22,23,26,28]. Studies suggest that elevated dietary salt levels contribute to immune system activation, resulting in the infiltration of pro-inflammatory immune cells into various organs [22,23,26,28–30]. Numerous investigations have affirmed that elevated salt levels contribute to macrophage polarization towards an M1 phenotype, while concomitantly suppressing the M2 phenotype [18,25–27]. Additionally, elevated salt levels have been associated with the differentiation of naïve T cells into Th17 cells through activation of the p38/mitogen activated protein kinase pathway [38,39]. The current study reveals that, despite HYD effectively lowering BP in SSHTN mice, the reduction in BP alone does not completely decrease all elevated pro-inflammatory immune cells involved in SSHTN. In male SSHTN+HYD mice, renal NK cells, CD4+TNFa+ Th1 cells, and Th17 cells, along with testicular CD4+IFNg+ Th1 cells remained elevated. SSHTN+HYD females exhibited a significant increase in renal M1 macrophages, DCs, NK cells, CD4+IFNg+ Th1 cells, and Th17 cells, as well as ovarian M1 macrophages. Furthermore, BP reduction did not lead to significant improvements in anti-inflammatory immune cells, including renal Tregs and gonadal M2 macrophages in either of the sexes. This implies that, while BP plays a crucial role in SSHTN, elevated salt levels may also contribute significantly to immune dysregulation and inflammation associated with SSHTN. This aligns with previous studies highlighting the multifaceted nature of SSHTN, where excessive dietary salt intake is recognized as a major factor in raising BP and contributing to end organ dysfunction.

The current investigation also found an increase in renal and gonadal lymphatic density in both male and female SSHTN mice, corroborating previous studies conducted in our laboratory [7,31]. HYD treatment did not completely rescue the compensatory increase in lymphatic density in these organs, emphasizing the persistence of the inflammatory microenvironment in SSHTN. BP reduction may not sufficiently target the local inflammatory responses or immune activation responsible for the observed increase in lymphatic vessel density in the kidneys and gonads. Moreover, a high salt diet is known to trigger vascular endothelial growth factor C (VEGF-C)-mediated lymphangiogenesis in the skin of mice and rats [40–42]. This raises the intriguing possibility that the increased renal and gonadal lymphatic density in SSHTN may be a result of high salt levels. As such, there is a need for a closer examination of the complex interplay of factors contributing to inflammation and lymphatic remodeling.

The role of systemic high blood pressure in causing renal injury is multifaceted, with several theories proposed by researchers [43–46]. One prominent mechanism involves the impairment of renal autoregulation, which denotes the kidney’s intrinsic ability to maintain constant renal blood flow and glomerular filtration rate (GFR) despite fluctuations in systemic blood pressure within a certain range [43,47]. Hydralazine, known for its systemic vasodilatory effects, indirectly impacts renal blood flow due to its ability to reduce overall blood pressure and vascular resistance [48]. When systemic blood pressure decreases as a result of hydralazine-induced vasodilation, renal blood flow might have increased as a compensatory mechanism to maintain kidney perfusion. This dynamic interplay between hydralazine and renal function is highlighted by the observed increase in urine excretion and fractional excretion of sodium (FENa) in male SSHTN+HYD mice. The observed increase in urine excretion and FENa in male SSHTN+HYD mice suggests that the vasodilatory and BP lowering effects of HYD improved renal sodium handling, by increasing the renal blood flow potentially contributing to the reduction in SBP. Furthermore, the improvement of gonadal function in both male and female SSHTN mice following the lowering of BP could be partially related to the modulation of pro-inflammatory immune cells and inflammation. This signifies the potential interplay between BP, the immune response, and reproductive health, emphasizing the broader implications of BP regulation for addressing reproductive complications in men and women with SSHTN. One of the limitations of our study is that, although the tail-cuff method is adequate for detecting notable variations in blood pressure, as documented in this study, employing a telemetry approach could offer greater accuracy in obtaining blood pressure values.

In conclusion, the current study demonstrates that decreasing BP with HYD generally mitigates pro-inflammatory immune cells and inflammation in the kidneys and gonads of male and female mice with SSHTN although differential effects were noted. However, lowering BP did not fully alleviate inflammation-associated lymphangiogenesis in the kidneys and gonads of either male or female SSHTN mice. The results of the current study suggest that lowering BP alone may not be adequate to completely resolve the end organ damage associated with SSHTN. These findings highlight the need for comprehensive management targeting both BP and dietary salt intake to effectively address SSHTN complications and associated tissue-specific responses.

## Clinical perspectives

- The link between SSHTN, elevated BP, and inflammation is well established. However, the specific contributions of a high salt diet versus an elevated BP to inflammatory mechanisms in SSHTN remains unclear.
- The current data suggest a nuanced impact wherein an elevated BP, in conjunction with salt, influences certain immune cell populations, while other immune cell populations exhibit alterations solely attributed to salt. This intricate interplay between high systemic pressure and salt underscores the complexity of their combined effects on the immune system.
- The findings contribute valuable insights for tailoring personalized therapeutic interventions in men and women with SSHTN, emphasizing the importance of understanding the mechanisms involved in the end organ damage associated with SSHTN.

## Supplementary Material

Supplementary Figures S1-S9 and Tables S1-S3

## Data Availability

All supporting data are included within the article.

## Abbreviations

BP: blood pressure
DC: dendritic cell
DPBS: Dulbecco’s phosphate-buffered saline
FBS: fetal bovine serum
FENa: fractional excretion of sodium
HTN: hypertension
HYD: hydralazine
IFN: interferon
IL: interleukin
L-NAME: nitro-l-arginine methyl ester hydrochloride
LYVE1: lymphatic vessel endothelial hyaluronan receptor 1
NK: natural killer
PBS: phosphate-buffered saline
PFA: paraformaldehyde
SBP: systolic blood pressure
SEM: standard error of the mean
SSHTN: salt-sensitive hypertension
Th1: T helper 1
Th17: T helper 17
Th2: T helper 2
Treg: regulatory T cell
VEGF-C: vascular endothelial growth factor C
