# Supplementary material for: Differential changes in end organ immune cells and inflammation in salt-sensitive hypertension: effects of lowering blood pressure
Source: Clin Sci (Lond). 2024 Jul 16;138(14):901–20. doi: 10.1042/CS20240698 (PMC11250109; doi:10.1042/CS20240698)
Supplement: Supplementary Figures S1-S9 and Tables S1-S3 [file CS-2024-0698_supp.pdf]

DATA SUPPLEMENT

Differential Changes in End Organ Immune Cells and Inflammation in Salt-Sensitive Hypertension: Effects of Lowering Blood Pressure

Authors and Affiliations

Shobana Navaneethabalakrishnan, Bethany L. Goodlett, Hannah L. Smith, Alyssa Cardenas, Asia Burns, Brett M. Mitchell

Department of Medical Physiology, Texas A&M University College of Medicine, Bryan, TX, U.S.A.

Corresponding Author

Brett M. Mitchell, PhD, ORCID: 0000-0002-2575-8761, brettmitchell@tamu.edu, 8447 Riverside Parkway, Medical Research Education Building II, Bryan, Texas, 77807, USA; Tel: +1 979 436 0751

Competing Interests

The authors declare that there are no competing interests associated with the manuscript.

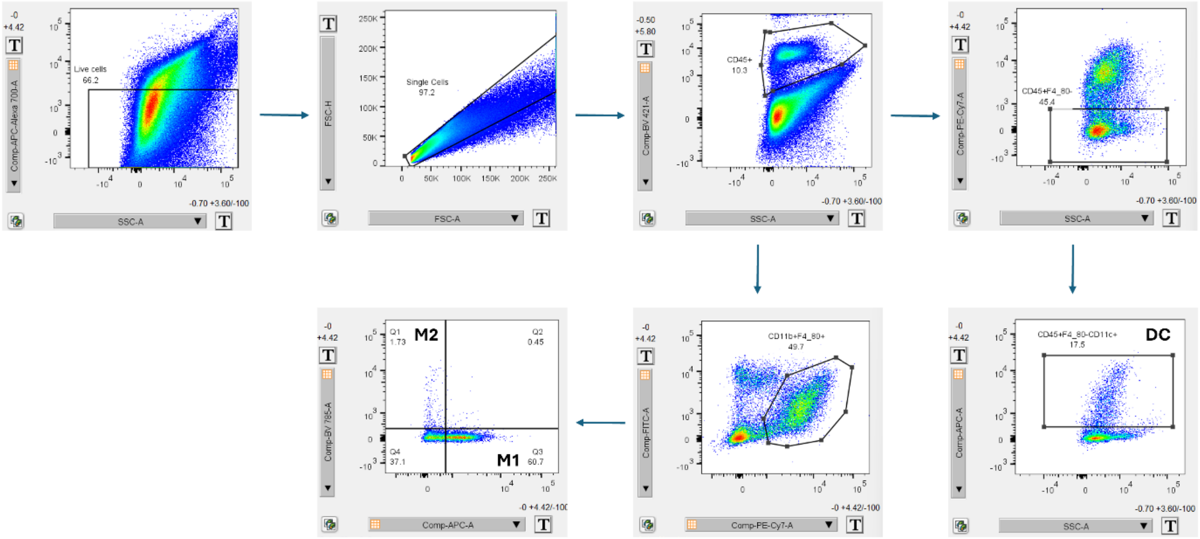

Supplemental Figure 1. Gating strategy for innate immune cells in the kidney. Cells were gated to select for live, singlet, CD45+ cells. Then, cells were analyzed for the markers shown.

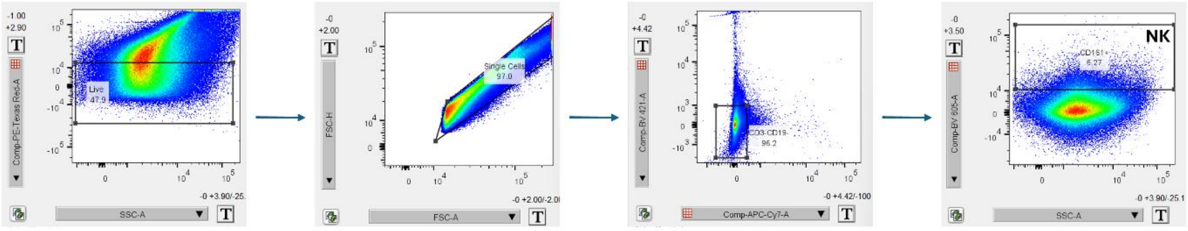

Supplemental Figure 2. Gating strategy for NK cells in the kidney. Cells were gated to select for live, singlet, CD45+ cells. Then, cells were analyzed for the markers shown.

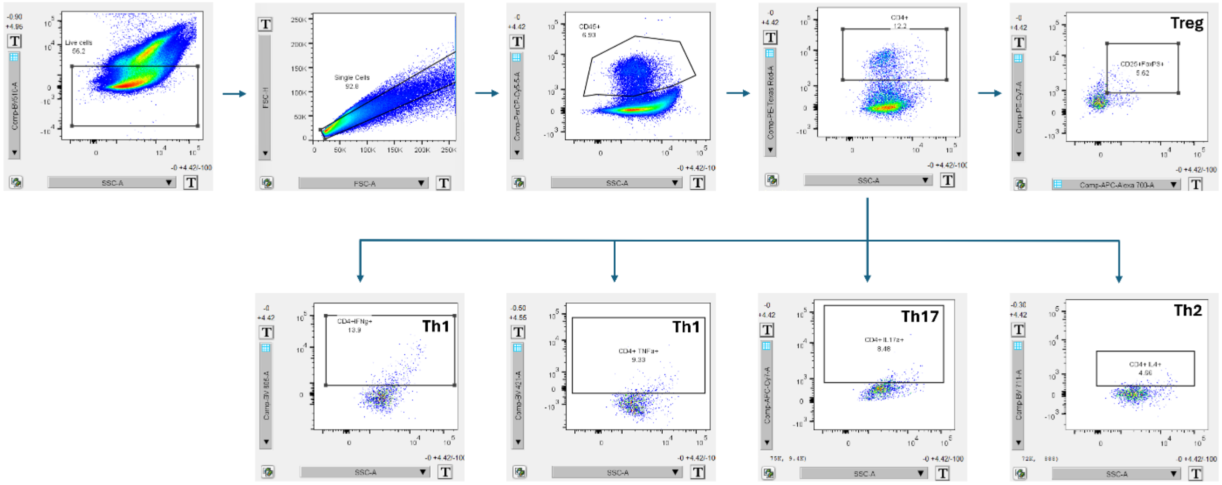

Supplemental Figure 3. Gating strategy for CD4-lineage T cells in the kidney. Cells were gated to select for live, singlet, CD45+ cells. Then, cells were analyzed for the markers shown.

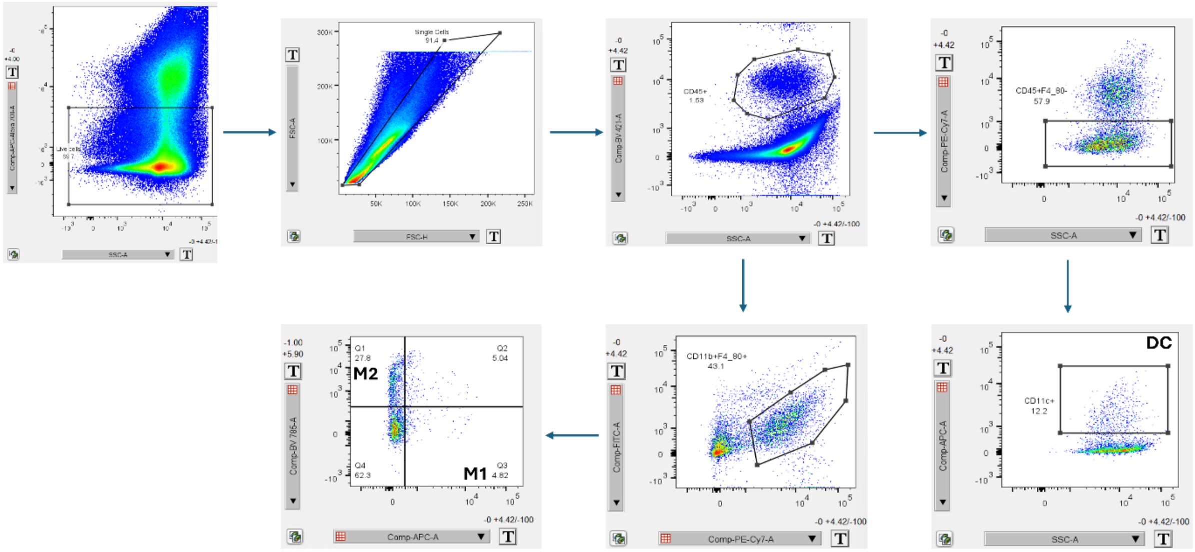

Supplemental Figure 4. Gating strategy for innate immune cells in the testes. Cells were gated to select for live, singlet, CD45+ cells. Then, cells were analyzed for the markers shown.

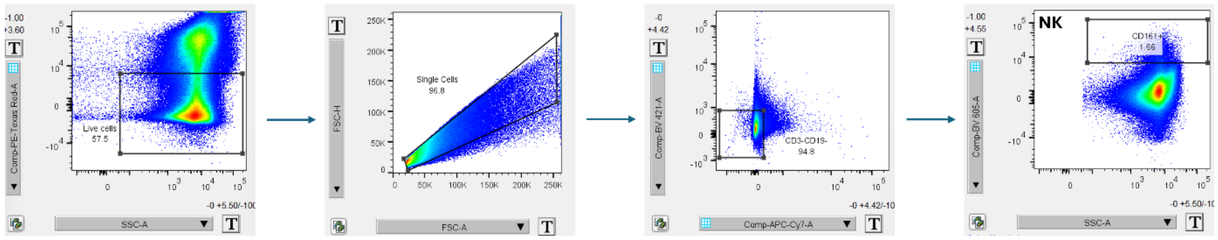

Supplemental Figure 5. Gating strategy for NK cells in the testes. Cells were gated to select for live, singlet, CD45+ cells. Then, cells were analyzed for the markers shown.

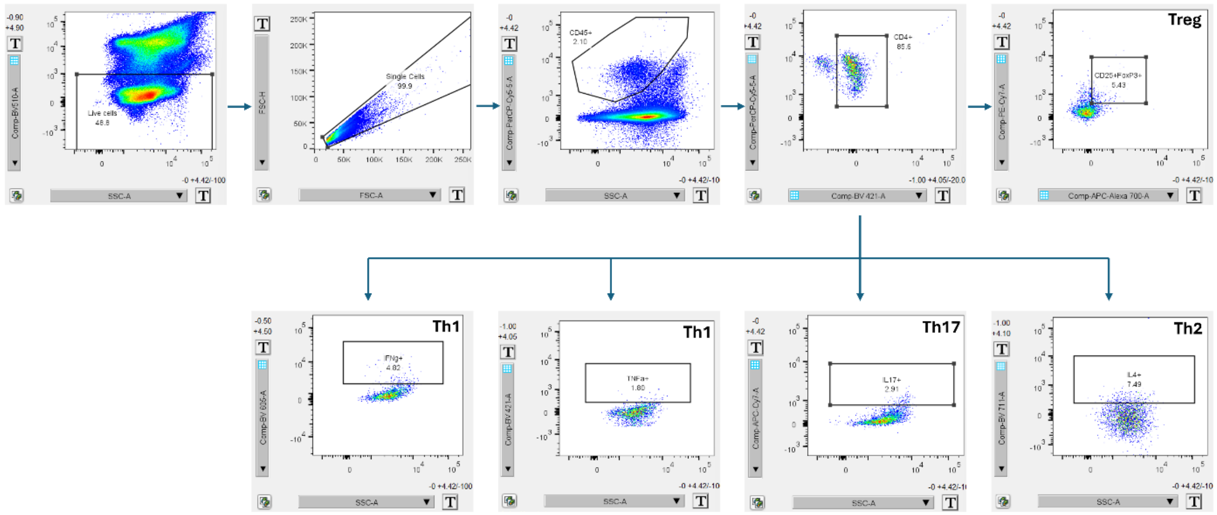

Supplemental Figure 6. Gating strategy for CD4-lineage T cells in the testes. Cells were gated to select for live, singlet, CD45+ cells. Then, cells were analyzed for the markers shown.

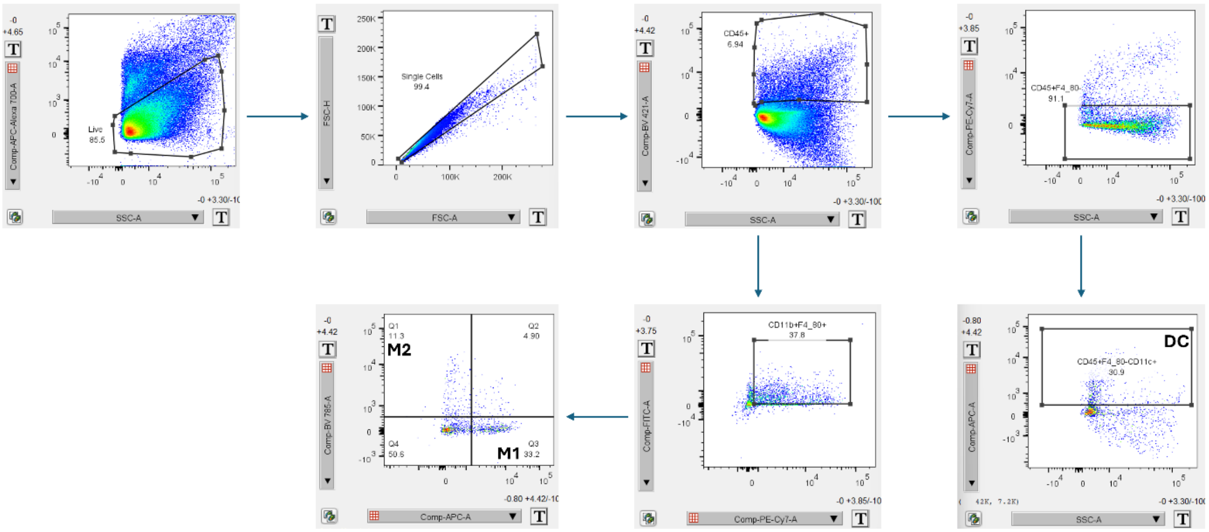

Supplemental Figure 7. Gating strategy for innate immune cells in the ovaries. Cells were gated to select for live, singlet, CD45+ cells. Then, cells were analyzed for the markers shown.

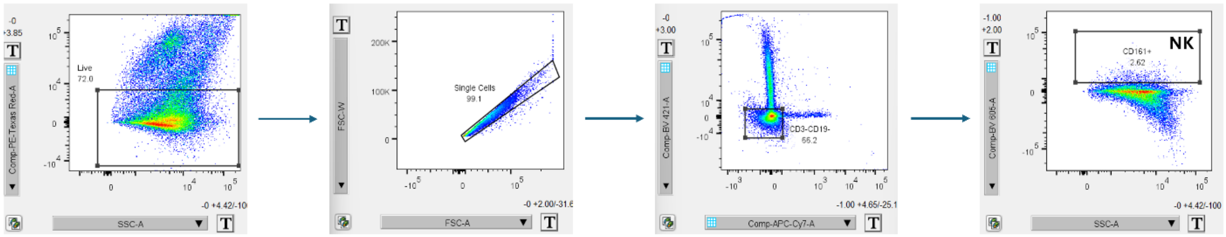

Supplemental Figure 8. Gating strategy for NK cells in the ovaries. Cells were gated to select for live, singlet, CD45+ cells. Then, cells were analyzed for the markers shown.

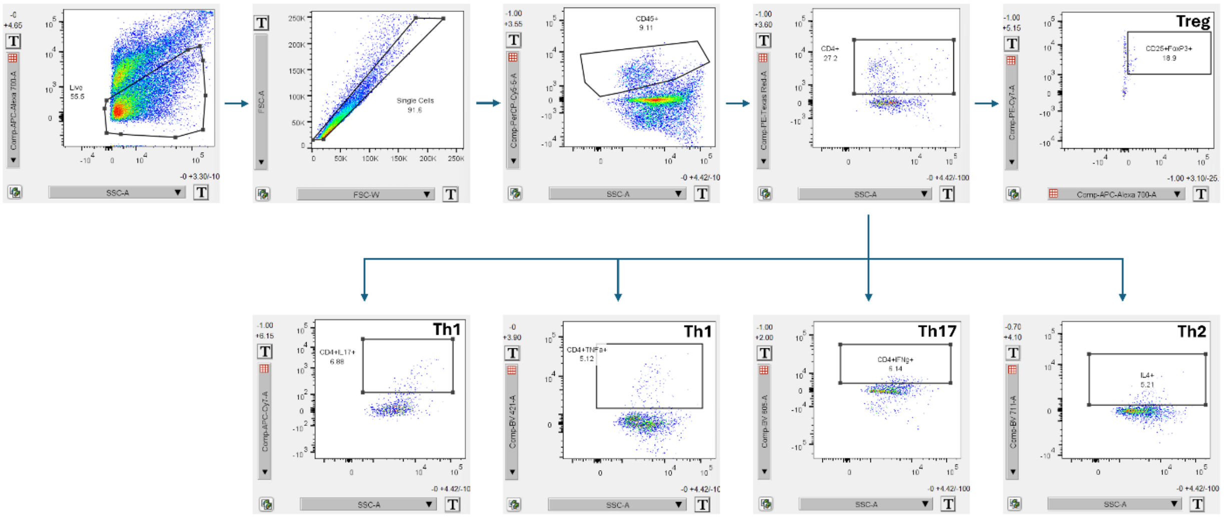

Supplemental Figure 9. Gating strategy for CD4-lineage T cells in the ovaries. Cells were gated to select for live, singlet, CD45+ cells. Then, cells were analyzed for the markers shown.

Online Table I. Flow cytometry antibody panel descriptions for mouse kidneys and gonads.

Panel	Innate Immune Cells					
**Fluorochrome**	BV421	FITC	APC	PE-Cy7	BV785	Ghost Dye Red 710
**Antigen**	CD45.2	CD11b	CD11c	F4/80	CD206	Live/dead
**Final Conc (µg/mL)**	2 (kidney & ovary)<br>4 (testis)	5 (kidney & ovary)<br>10 (testis)	2 (kidney & ovary)<br>4 (testis)			
**Dilution Factor**	1:100 (kidney & ovary)<br>1:50 (testis)					1:400
**Clone**	104	M1/70	N418	BM8	C068C2	
**Manufacturer**	BD	BioLegend	BioLegend	BioLegend	BioLegend	Tonbo

Panel	Natural Killer Cells			
**Fluorochrome**	Pacific Blue	BV605	APC-Cy7	Zombie Red
**Antigen**	CD3e	CD161	CD19	Live/dead
**Final Conc (µg/mL)**	2 (kidney & ovary)<br>4 (testis)			
**Dilution Factor**	1:100 (kidney & ovary)<br>1:50 (testis)			1:200
**Clone**	500A2	PK136	1D3	
**Manufacturer**	BD	BioLegend	BD	BioLegend

Panel	CD4-Lineage T Cells			
**Fluorochrome**	BV421	PE-Cy5	PE-Cy7	Ghost Dye Violet 510
**Antigen**	CD45.2	CD4	CD25	Live/dead
**Final Conc (µg/mL)**	2 (kidney & ovary)<br>4 (testis)			
**Dilution Factor**	1:100 (kidney & ovary)<br>1:50 (testis)			1:400
**Clone**	104	RM4-5	PC61	
**Manufacturer**	BD	BioLegend	BioLegend	Tonbo

Panel	CD4-Lineage T Cells (Intracellular)				
**Fluorochrome**	BV605	BV711	AlexaFluor 700	BV421	APC-Cy7
**Antigen**	IFNg	IL4	FoxP3	TNFa	IL17a
**Final Conc (µg/mL)**	2 (kidney & ovary)<br>4 (testis)				
**Dilution Factor**	1:100 (kidney & ovary)<br>1:50 (testis)				
**Clone**	XMG1.2	11B11	FJK-16s	MP6-XT22	TC11-18H10.1
**Manufacturer**	BioLegend	BioLegend	eBioscience	BD	BioLegend

Abbreviations: APC = allophycocyanin; BV = brilliant violet; FITC = fluorescein isothiocyanate; PE = phycoerythrin; PerCP-Cy5.5 = peridinin chlorophyll protein complex cyanine 5.5

Online Table II. Immune cell antibody staining profiles.

**Immune cell**	**Phenotype**
M1 macrophages	CD45+ CD11b+ F4/80+ CD11c- CD206+
M2 macrophages	CD45+ CD11b+ F4/80+ CD11c+ CD206-
Dendritic cells	CD45+ F4/80- CD11c+
NK cells	CD45+ CD3- CD19- CD161+
IFNg+ Th1 cells	CD45+ CD3+ CD4+ IFNg+
TNFa+ Th1 cells	CD45+ CD3+ CD4+ TNFa+
Th17 cells	CD45+ CD3+ CD4+ IL17+
Tregs	CD45+ CD4+ CD25+ FoxP3+
Th2 cells	CD45+ CD3+ CD4+ IL4+

Online Table III. Primer sequences for qRT-PCR analysis of murine renal and gonadal tissue.

Target	Forward (5' to 3')	Reverse (5' to 3')
Ar	CCCTGAGGCCGCTAACATAG	GGGCTTGAGGAGAACCATCC
Ccl19	GGGGTGCTAATGATGCGGAA	CCTTAGTGTGGTGAACACAACA
Ccl21	CCCTGCTTCAACCATTACATCTGC	CCTGCTGTCTCCTTCCTCATTCC
Ccr7	TGTACGAGTCGGTGTGCTTC	GGTAGGTATCCGTCATGGTCTTG
Cldn11	TTGCTCTTTCCTCGGGCATT	CCCAATCCACACCCAAGTCA
Cyp11a1	GGGGACAGTATGCTGGCTAA	ACGTAGGGCTCAGGAAAGGT
Cyp17a1	TGGAGGCCACTATCCGAGAA	CACATGTGTGTCCTTCGGGA
Cyp19a1	TCACTCTACTAACTCAAGGGCG	GGGAGGCTCAGGTTCTGTTC
Era	AATTCTGACAATCGACGCCAG	GTGCTTCAACATTCTCCCTCCTC
Fshr	GGTCTATTCCCTGCCCAACC	AGGGAGCTTTTTCAAGCGGT
Hsd3b1	CAGGAGAAAGAACTGCAGGAGGTC	GCACACTTGCTTGAACACAGGC
Hsd17b1	AATTGAACGCTGTGGGTGCT	GAATGGCAGTCCCATCAAGC
Icam	GTGATGCTCAGGTATCCATCCA	CACAGTTCTCAAAGCACAGCG
Ifng	TCAAGTGGCATAGATGTGGAAGAA	TGGCTCTGCAGGATTTTCATG
Il1b	GCCACCTTTTGACAGTGATGAG	GACAGCCCAGGTCAAAGGTT
Il6	GAGGATACCACTCCCAACAGACC	AAGTGCATCATCGTTGTTCATA
Il10	GCTCTTACTGACTGGCATGAG	CGCAGCTCTAGGAGCATGTG
Il17	TTTAACTCCCTTGGCGCAAAA	CTTTCCCTCCGCATTGACAC
Inhba	AAATCAGAACGCCTCCGCTA	TCCCGAGTGTAGAGTTCGGT
Inhbb	AGGCCAGCGGATCAGTTTTA	CAGGCCACTCGAAGGATTGT
Lhr	ACGAGACGCTTCATCACTCTG	GATGGCATGTCTCAGCCTCA
Lyve1	CTGACAAGCAGTTTCAGGCTTGGT	TTCAGCCCACACTCCGCTATACAT
Nos2	GTTCTCAGCCCAACAATACAAGA	GTGGACGGGTCGATGTCAC
Ocln	CCCCTCTTTCCTTAGGCGACA	AGGCTCCCAAGATAAGCGAAC
Pdpn	ACCGTGCCAGTGTTGTTCTG	AGCACCTGTGGTTGTTATTTTGT
Prox1	CTCTTGCCTCGCTATCCCC	CACAGTCCCACTGACGTACC
Scgb1b24	GCTCCTGCATTCAGGGGTAT	ACATACTCTTCTGAGGTCCTGTG
Star	GAACGGGGACGAAGTGCTAA	TGGTCTACCACCACCTCCAA
Trf	AGAACCGCTGGTTGGAACAT	GCGCAGCCTTGACTGAAAAA
Tjp1	AGACGCCCGAGGGTGTAG	TGGGACAAAAGTCCGGGAAG
Tnfa	GAGAAAGTCAACCTCCTCTCTG	GAAGACTCCTCCCAGGTATATG
Ubc	GCCCAGTGTTACCACCAAGAAG	GCTCTTTTTAGATACTGTGGTGAG
Vcam	AGTTGGGGATTCGGTTGTTCT	CCCCTCATTCCTTACCACCC
Vegfc	CAGTGTCAGGCAGCTAACAAG	GAAGGTCCACAGACATCATGGAA
Vegfd	TGGCAAGACTTTTGAGCTTCAA	AAATCGCGCACTCTGAGGA
Vegfr2	GCCCTGCTGTGGTCTCACTAC	CAAAGCATTGCCCATTCGAT
Vegfr3	ATCAGAAGATCGGGCGCTGTTGTA	TGTGTCATGTCCGCCCTTCAGTTA

All sequences were verified through National Center for Biotechnology Information Primer-BLAST and single products were confirmed with a melting point dissociation step post amplification.

Ar, androgen receptor; Ccl19, chemokine ligand 19; Ccl21, chemokine ligand 21; Ccr7, C-C chemokine receptor type 7; Cldn11, claudin 11; Cyp11a1, cytochrome P450 side chain cleavage; Cyp17a1, cytochrome P450 17α-hydroxylase; Cyp19a1, cytochrome P450 aromatase; Era, estrogen receptor alpha; Fshr, follicle stimulating hormone receptor; Hsd3b1, 3-beta hydroxysteroid dehydrogenase; Hsd17b1, 17-beta hydroxysteroid dehydrogenase; Icam, intercellular adhesion molecule; Ifng, interferon gamma; Il1b, interleukin 1 beta; Il6, interleukin 6; Il10, interleukin 10; Il17, interleukin 17; Inhba, inhibin beta a subunit; Inhbb, inhibin beta b subunit;

Lhr, luteinizing hormone receptor; Lyve1, lymphatic vessel endothelial hyaluronan receptor 1; Nos2, nitric oxide synthase 2; Ocln, occludin; Pdpn, podoplanin; Prox1, prospero homeobox 1; Scgb1b24, secretoglobin family 1B, member 24; Star, steroidogenic acute regulatory protein; Trf, transferrin; Tjp1, tight junction protein-1; Tnfa, tumor necrosis factor alpha; Ubc, ubiquitin; Vcam, vascular cell adhesion molecule; Vegfc, vascular endothelial growth factor C; Vegfd, vascular endothelial growth factor D; Vegfr2, vascular endothelial growth factor receptor 2; Vegfr3, vascular endothelial growth factor receptor 3.
